# In Vitro Human Dermal Absorption Studies on Pesticides in Complex Mixtures: Investigation of Guidance Criteria and Possible Impact Parameters

**DOI:** 10.3390/toxics12040248

**Published:** 2024-03-28

**Authors:** Christina Pieper, Nadine Engel, Korinna Wend, Carsten Kneuer, Sabine Martin

**Affiliations:** Department of Pesticides Safety, German Federal Institute for Risk Assessment (BfR), Max-Dohrn-Str. 8-10, 10589 Berlin, Germanykorinna.wend@bfr.bund.de (K.W.);

**Keywords:** dermal absorption, in vitro, human skin, pesticides, biocides, risk assessment, pro rata approach, default values, in silico

## Abstract

Pesticides must not pose unacceptable risks to human health, so risk assessments are conducted before products are authorised. Dermal exposure is often the main route of intake, so estimating realistic and trustworthy dermal absorption values is crucial for risk assessment. Although there are agreed test guidelines for in vitro dermal absorption studies, not every product is tested due to cost reasons. The present dataset consists of 945 individual in vitro experiments on the dermal absorption of human skin with 179 active substances of pesticides in 353 different mixtures, including concentrates and dilutions. The dataset was evaluated to identify the possible impacts of experimental conditions and physico-chemical properties on dermal absorption. The dataset was also analysed to assess the appropriateness of the pro rata correction for untested dilutions, and the set concentration cut-off to decide on the dilution status for choosing a default value on dermal absorption. The study found that the implementation of specific guidelines improved the harmonisation of study conduct, with support for approaches such as pro rata correction and default values. Further analysis of the specific co-formulants may identify influencing factors that may be more important than the experimental variables.

## 1. Introduction

Plant protection products and biocidal products should not pose unacceptable risks for the human health; therefore, risks resulting from specific applications are characterised and suitable risk mitigation measures are assigned if required before products are authorised, e.g., in the European Union (EU), according to Regulation (EC) No. 1107/2009 [1] and Regulation (EU) No. 528/2012 [2]. Risk assessment comprises hazard identification (e.g., the derivation of toxicological reference values, classification and labelling), exposure assessment and risk characterisation. The dermal exposure route is often the main route of entry following the intended use of plant protection and biocidal products. Thus, this parameter mainly controls the systemic exposure level of substances, and the estimation of dermal absorption represents a crucial step in the human risk assessment of plant protection products and biocidal products for operators, workers, bystanders, and residents/general population. Information on the skin absorption of the active substance and other toxicological relevant compounds is a data requirement under biocidal and/or plant protection product regulation (Regulation (EC) No. 528/2012; Regulation (EC) No. 1107/2009) when dermal exposure occurs to the product and default assumptions do not result in an acceptable risk. Due to the major impact on the authorisation decisions of pesticides and necessary risk mitigation measures, realistic and trustworthy dermal absorption values are needed to enable reliable risk assessments of the intended uses of pesticides.

In the EU, for plant protection products and biocidal products, the assessment of dermal absorption is performed using a tiered approach recommended by the European Food Safety Authority (EFSA) Guidance on dermal absorption [3]. In the first tier, default values of dermal absorption can be used if no specific data on dermal absorption are available. If the systemic exposure exceeds the relevant toxicological reference value, in vitro data with human skin or rat skin (tier 2), or existing in vivo data with rats (tier 3) should be used if available. If, at any stage of the tiered approach, the predicted exposure is below the systemic reference dose, e.g., the Acceptable (Operator) Exposure Level (A(O)EL), no further assessment or testing is required. However, if the exposure prediction is not below the reference value, and no suitable data on dermal absorption are available, in vitro testing with a representative formulation according to the Organisation for Economic Co-operation and Development (OECD) Test Guideline (TG) 428 [4], using human skin is accepted as the stand-alone method in the EU and should be performed preferably.

Besides the test guidelines for a harmonised study conduct (e.g., OECD TG 428 for in vitro testing), the OECD published documents to enhance the harmonised interpretations of experimental data from dermal absorption studies (OECD Guidance Document No 28 [5] and Guidance Notes No 156 [6,7]). EFSA also published the Guidance on Dermal Absorption, which recommends additional rules for conducting and evaluating studies, particularly to improve the reliability of dermal absorption assessments for active substances used in plant protection products in regulatory frameworks [3,8]. This guidance on dermal absorption is applied for biocides as well. However, in many cases, the respective formulation is not tested itself, but dermal absorption studies are performed with formulations different from the plant protection product or biocidal product applied for. Thus, in regulatory risk assessment, the similarity of formulations with different co-formulants has to be assessed by estimating the possible impact of the co-formulants on dermal absorption. EFSA [3] provides rules to extrapolate the data to different formulations and exposure conditions. Generally, pesticides are formulated to adhere to the leaf or penetrate the leaf surface (or better: cell membranes) in order to be systemically available to affect target organisms. Hence, for humans, the systemic exposure assessment via the dermal route is likely to be more critical for pesticides than, e.g., for cosmetics, which are formulated to stay on, or in, the upper layers of the skin without the aim of causing harm to organisms. Thus, EFSA [3] recommends study quality criteria for pesticides going beyond those recommended by OECD TG 428 [4] or respective OECD guidance documents (e.g., recovery of ≥95 to ≤105%, donor and replicate number (eight samples originating from four donors), addressing variability within results) to ensure high quality of data and prevent the underestimation of dermal absorption. This also includes the demonstration of reasons for possible losses. For volatile or potentially volatile compounds, measures should be taken to prevent loss (e.g., charcoal filter occlusion).

As reported in the literature, there are many factors influencing the penetration process of substances across the skin barrier [9]. The penetrated dose may be affected by the physico-chemical parameters of the penetrant, condition of the skin or skin sample, experimental conditions and other factors, as well as the formulation tested [10,11,12,13,14]. Theoretically, the measurement of the penetration of substances through the skin barrier may be supplemented by a prediction tool based on specifically gathered data on the dermal absorption of related substances and experimental conditions. Simple models are available from the literature to predict the dermal absorption of chemicals based on physico-chemical parameters of the chemical, e.g., octanol–water partition coefficient and molecular weight [15,16,17]). However, in the literature, only models with parameters to predict the dermal absorption of chemicals in a defined solvent/mixture and simple vehicle are described. In particular, pesticide products are formulated to ensure or increase the efficacy of the active substance. Eleftheriadou et al. [18] investigated the potential for the prediction of the dermal absorption of substances in complex mixtures of plant protection products using several published models developed for single substances in simple solutions. The models generally failed to accurately predict experimental values.

Other published models examine whether the incorporation of a mixture factor concerning the physico-chemical properties of the mixture improve predictability of the models [19,20,21]. Nonetheless, information on reliable in silico models for the prediction of dermal absorption from complex mixtures and considering mixture effects are limited, especially for highly sophisticated pesticides and biocides.

Kneuer et al. [22] investigated available in silico models for the prediction of absorption across the skin. For this, suitable models potentially relevant to the regulatory assessment of pesticides were selected from the literature to compare predicted dermal absorption values versus measured data of plant protection products using a large dataset of in vitro dermal absorption studies. While some models were based on the physico-chemical, quantum-chemical, or structural properties of the substance only, some of the selected models took limited parameters of the formulation into account. Models which took formulation parameters into account incorporated a mixture factor based on the physico-chemical properties of the main constituents in the formulation. Nonetheless, the specific combination of co-formulants was not part of the selected models. Kneuer et al. [22] highlighted that one model is worthy for further investigations to estimate the dermal absorption value in the absence of adequate experimental data, but it was noted that the influence of physico-chemical properties had to be investigated further. Furthermore, none of the selected models took into account the possible co-formulant’s effect on the stratum corneum. Further knowledge concerning the influence of exposure conditions and formulation parameters is needed to meet current challenges in regulatory risk assessment.

The Food and Agriculture Organisation of the United Nations (FAO)/World Health Organisation (WHO) [23] terms 65 formulation codes for pesticide formulations. In general, co-formulants are mixtures themselves, and are very variable in their composition. The German directory for co-formulants registered in Germany and used in approved plant protection products [24] lists 1654 co-formulants with a known composition. Detailed information on the composition of pesticide products, from the type of formulation and co-formulants to the level of the single substances of co-formulants, highlights the diversity of pesticide formulations. Moreover, it is known that the presence of co-formulants in pesticide products can alter the toxicological and toxicokinetic profile, including the penetration of active substances across the skin [3,11]. Thus, for risk assessment, it is necessary to consider vehicle effects and the composition of the formulation of the active substance. Most of the existing in silico models only consider the dermal absorption of one penetrant in simple aqueous vehicles and do not consider complex pesticide product compositions as described above. However, due to the broad variety and complexity of commercial plant protection products and biocidal products, it is challenging to identify reasonable factors of the mixture which play an important role in influencing the dermal absorption of the active substance.

As described above, dermal absorption is a critical element in the risk assessment of pesticides for the concerned population (operators, workers, bystanders and residents). Although agreed test guidelines exist for the performance of in vitro dermal absorption studies, not every product or dilution is tested for cost reasons. Therefore, it is often necessary to investigate whether data from tested products can be transferred to untested ones. Furthermore, there is a growing interest in the potential to use in silico tools for the prediction of dermal absorption, but for complex formulations, further research is needed.

The identification of relevant parameters influencing dermal absorption might enable the development of recommendations to improve the risk assessment of active substances in different formulations, as well as to read across untested formulations in the field of pesticides.

Studies on dermal absorption, which were submitted to the German Federal Institute for Risk Assessment (BfR) for the authorisation of plant protection products, or active substance evaluation up to October 2018, were considered for the BfR database dermal absorption. The present dataset of in vitro experiments on dermal absorption consists of 945 individual dermal absorption in vitro experiments on human skin with 179 active substances of pesticides in 353 different mixtures (=formulations), including concentrate and dilutions, if relevant. It contains information on study conduct parameters, the physico-chemical properties of the active substance and the formulation.

The permeation rate of compounds through the skin can be described either as an absolute amount, in analogy to Fick’s diffusion equation, or as percentage of the applied dose [4]. For the regulatory risk assessment of pesticides in the EU, the percentage absorbed relative to the external dose is required as a descriptor of absorption [3]. Exposure scenarios for which a risk assessment for operators, workers, bystanders, residents/consumers is performed vary widely with regard to the extent and duration of exposure as well as the exposed skin area. Exposure models are employed to estimate the external exposure in absolute amounts per person per day (i.e., µg/person/d), and a conversion factor is needed to calculate the resulting systemic (or internal) exposure. This conversion factor is obtained from in vitro dermal absorption studies performed using representative exposure situations and is expressed as a percentage of the applied dose. Unfortunately, test conditions cannot always be fully equivalent to those encountered in real-life exposures. Therefore, this publication, with a focus on pesticide risk assessment retrospectively, examines the influence of experimental and other parameters on this conversion factor, i.e., the relative dermal absorption in percent, as used in the specific regulatory context.

The dataset is described, e.g., by the chemical space or the distribution of dermal absorption. The mass balance distribution of the dataset is evaluated to assess whether the recommended mean recovery criteria of above or equal to 95% ([3]) can be met, and whether the development of additional guidelines for conducting the studies has had an impact on quality.

Based on the considerations above, the dataset was analysed to identify the possible impacts of experimental conditions in in vitro dermal absorption testing as well as the influence of certain physico-chemical properties of the active substance or the formulation type on dermal absorption, being relevant for bridging of data.

Due to different applications, often not all dilutions recommended on the product label are tested. The pro rata approach described in [3] is based on the assumption that the percentage dermal absorption is usually inversely proportional to substance concentration. The present dataset is evaluated regarding the occurrence of non-inversely related percentage dermal absorption and the applicability of the pro rata correction [3] as the dataset contains several experiments for the concentrate and two or more dilutions. Furthermore, EFSA proposes default values for untested products based on the formulation type and dilution status (active substance concentration) [3]. The present dataset was analysed to estimate the impact of the set threshold active substance concentration to distinguish concentrate products from dilutions.

## 2. Materials and Methods

### 2.1. Dataset and Experimental Conditions of Studies

**Figure 1:**
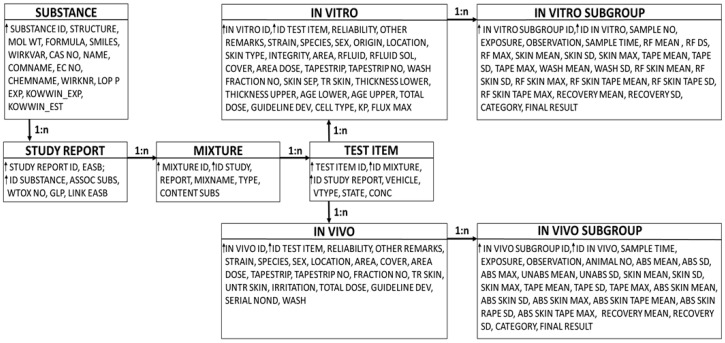
Schematic view of the BfR relational MySQL database dermal absorption. Each box represents a table (entity) containing the fields named in the lower part of each box. Each table’s primary key is represented by the first of the listed variables. Both primary and foreign keys are marked with an arrow (↑). The cardinality (1:n) describes the relationship between the tables (e.g., one entry in the table STUDY REPORT is associated with exactly one entry in the table SUBSTANCE, but can be associated with many (n) entities in the table of the type MIXTURE).

The BfR database on dermal absorption is a relational MySQL database, and the structure is shown in Figure 1.

The software used for creating, editing and querying was Instant JChem software (version: 19.1.0, Chemaxon Ltd., Budapest, Hungary). The information on the experimental dermal absorption data of pesticide-active substances was collected from studies on dermal absorption submitted for the authorisation of plant protection products and biocidal products in Germany, or active substance evaluation in the EU. All studies were performed in various laboratories according to OECD TG 428 [4] and good laboratory practice to reduce distinct experimental differences. The current assessment focuses only on in vitro data on human skin. The human in vitro dermal absorption data comprised information on the tested active substance (name, molecular weight (MW), concentration), the tested formulation (formulation/mixture type, category of formulation according to EFSA [3], experimental details (exposure time, observation time, composition of receptor fluid, used skin type (preparation), cover (occlusive or non-occlusive), cell type (static or dynamic)), experimental results (measured absorption in different compartments such as the receptor fluid, skin preparation or the tape strip fractions as well as skin wash including donor fraction).

Additionally, the following physico-chemical parameters of the active substances were included in the database, based on experimental values or calculated with EPISuite™ (US EPA 2012) or the Instant JChem calculator plugins: logarithm of octanol/water partition coefficient (LogK_OW_), molar refractivity, negative base-10 logarithm of the acid dissociation constant (pKa), hydrogen bond acceptor count (HbA), hydrogen bond donor count (HbD), Balaban index (Balaban distance connectivity of the molecule (average distance sum connectivity)), topological polar surface area (TpSA), Van der Waals surface area (v-d-W SA), logarithm of distribution coefficient (LogD), molar polarisability.

The calculations were based on structure information with simplified molecular-input line-entry system (SMILES) codes. Experimental data on solubility in octanol and pKa values were collected, when available, from published EFSA conclusions, Registration Reports, Assessment Reports and Risk Assessment Committee (RAC) opinions. Where available, experimental values were preferred to calculated values. The dataset used for analysis is available in the Supplementary Material (refer to S1).

The recovery is one valuable parameter in dermal absorption studies to verify the reliability of the study in terms of experimental conditions and the appropriateness of the analytical detection method. Per definition, recovery is the mass balance of the applied test substance found in the different compartments for dermal absorption. A well-conducted study should demonstrate the cause of the losses, e.g., in the case of possible volatility, by using a specific test design.

To meet the criteria of reliable in vitro dermal absorption studies and to ensure the high quality of data, only experiments with mean recovery of 90–110% were listed in the dataset and considered for further analysis. The range of 90–110% recovery represents the recovery limits recommended by the respective OECD documents [4,5,6]. However, the recommended mean recovery of 95–105% according to EFSA [3] was not applied to prevent the skewing of data, and to perform several analyses as described below. In general, however, it should be noted that the reasons for such low mass balances should be further investigated in a study as described above. Subsequently, the dataset presented here consists of 945 individual dermal absorption in vitro experiments (mean values of compartments over replicates) for 179 active ingredients of pesticides in 353 different mixtures (=formulations). If relevant for the specific application of the pesticides, the concentrate and the respective dilution(s) were tested in the dermal absorption studies.

The dataset covers exposure times starting from 6 h until 48 h exposure of the skin sample with the test preparation, but mainly 8 h, 6 h or 24 h exposure experiments. The general exposure time relevant for non-dietary exposure assessment is 6 to 10 h; however, experiments with longer exposure durations were kept in order to also evaluate the respective impact. After exposure, the test preparation was washed off with cotton swabs and washing fluid (water, soap solution or ethanol–water mixtures). Moreover, the data on human skin samples include primarily dermatomed (split-thickness) skin but also isolated epidermis or full-thickness skin samples. Further, exposure conditions covered by the experiments were static, static stirred or dynamic (flow-through) exposure cell types and occlusive, semi-occlusive or non-occluded finite dose conditions. In 85.5% of all experiments, tape stripping with 1 to 20 tape strips was used to remove the upper *stratum corneum* from the skin.

For the risk assessment of pesticides, the total percentage penetration of a compound into (including the skin bound portion), and across, the skin was considered. Thus, the amount of dermal absorption was consistently calculated as percentage dermal absorption in agreement with EFSA [3]; although, it is noted that this approach has some pitfalls because the percentage absorption is generally not independent of skin-loading conditions [25]. The mean percentage dermal absorption was calculated as a sum of the mean replicate values of the amount in receptor fluid plus the amount in receptor chamber washes, the amount in skin samples and the mean amount in tape strips, if applicable, as these values are presented in the dataset. Normally, according to EFSA [3], all tape strips can be excluded when (I) the sampling period was 24 h and over 75% of the total absorption was reached within half of the duration of the total sampling period. Otherwise, when (II) the sampling period was less than 24 h or less than 75% of the total absorption occurred within half the time of study duration, all tape strips excluding tape strips 1 and 2 were considered absorbed. In order to treat each study result equally in the present analysis, tape strips were not excluded. Furthermore, missing amounts, identified with low recovery (<95%), were not addressed here because the dataset comprises mean replicate values instead of single replicates; the normalisation or addition of missing amounts are addressed during risk assessment and would mislead the outcome of the presented analysis. Additionally, the calculation of final dermal absorption values changed over years and, thus, cannot be taken into account for the analysis of the described dataset. The human in vitro study reports at issue here describe several experiments with one or more pesticide formulations at different concentrations or experimental conditions. Each experiment gives the endpoint of percentage dermal absorption calculated based on a minimum of four replicates per experiment (in only one experiment, three replicates were applied).

### 2.2. Descriptive Statistics

The experimental values and factors are based on random variations which require statistical analysis. Mean values of dermal absorption were plotted against other parameters, rather than the individual data points from each tested concentration, because the dataset contains the mean values of replicates of several analysed compartments. Therefore, no identification of any individual outlying data point was performed, and, thus, no potential individual outliers were removed from the data. The percentage dermal absorption values based on mean values were calculated as described in Section 2.1.

Analysis aimed to determine if any influencing parameter that relates to dermal absorption can be identified. The parameter could either be an active substance-related factor (physico-chemical property), related to co-formulants represented as formulation type, or related to exposure and experimental conditions (i.e., study parameters). The majority of the experiments resulted in rather low dermal absorption values. As the percentage dermal absorption values do not fit to a normal distribution, the data was log-transformed to achieve nearly normally distributed data. General statistical analysis was conducted with IBM SPSS Statistics (version 21), or with R (R Core Team 2020).

The covered chemical space of the dataset was described by molecule complexity (molecular weight) vs. molecule hydrophobicity as LogP. Additionally, the frequency distribution of the dermal absorption values in the dataset was presented.

The presented dataset was analysed to evaluate the distribution of the mass balance. The presented dataset comprises experiments with mean recovery of 90–110%, ensuring the high quality of data. Hence, other studies with lower mass balances are not covered.

The dataset consists of studies submitted to the BfR from the years 1996 to 2018. While the OECD TG 428 was published in 2004 [4], several guidance documents were published in 2004 [5], 2011 [6] and 2012 [8] (revised in 2017, [3]) for the conduct of dermal absorption studies on pesticides. The dataset was examined to see to what extent the recovery criteria of the EFSA Guidance document on dermal absorption [3] of 95–105% were met.

The possible effects of the formulation on dermal absorption were evaluated considering the dilution status (concentrate or dilution), the different formulation types as provided in the study report, and the four formulation categories, (1) organic solvent-based, (2) water-based/dispersed, (3) solid, (4) other, in analogy to the EFSA Guidance document on dermal absorption [3]. Furthermore, the same sub dataset was used to investigate the robustness of the general assumption that the percentage dermal absorption of an active substance increases with lower concentration, as described by Buist et al. [26]. Studies with at least two dilutions were evaluated regarding the course of absorption, depending on the active substance concentration, to conclude on the general assumption of an inverse relationship of absorption and dermal loading. Besides the effects of formulation types or formulation categories, the possible impact of the experimental parameters, (1) skin type, (2) state of occlusion, (3) cell type and (4) skin area, on dermal absorption were plotted against the log-transformed percentage dermal absorption (log%DA). In addition, the dataset was examined with regard to the contribution of exposure time to the average absorption.

### 2.3. Linear Regression of the Physico-Chemical Parameters of the Active Substances

The correlation between dermal absorption and selected physico-chemical parameters of the active substances in the dataset was analysed. Further, linear regression analysis was performed to identify a possible linear relationship between the dermal absorption and selected physico-chemical parameters of the active substances contained in the dataset.

The analysis was performed with the R statistics program (RStudio, version 1.3.1093, © 2024–2020 RStudio, PBC). Using ‘linear model’, the assumed linear relationship between the endpoint of log%DA and influencing parameters (the physico-chemical properties of the active substance) was modelled. *p*-values of *t*-test statistics were consulted for statistical significance. Additionally, a stepwise embedding of parameters in the regression model was performed. The Akaike information criterion (AIC) is a metric that is used to compare the fit of several regression models. Lower scores of AIC can indicate a better-fit model, relative to a model fit with a higher AIC. The AIC is an estimator of in-sample prediction error.

The mean log%DA and the following physico-chemical parameters were analysed (see also 2.1 for details): molecular weight (MW), logK_OW_, molar refractivity, Hbond acceptor count, Hbond donor count, TpSA, Van der Waals surface area, logD, molecular polarizability, receptor medium, log transformed concentration (logConc).

Although the receptor medium used in in vitro dermal absorption studies is not a physico-chemical property of the active substance, it is dependent on its properties and probably modified due to the solubility of the active substance. Due to the large number of different but similar receptor fluids used, these were divided into 5 groups: water with less than 50% ethanol (n = 68), water with 50% ethanol (n = 368), water/saline/culture medium (n = 132), water/saline/culture medium with bovine serum albumin (BSA, n = 189), water/saline/culture medium with surfactant (182). For comparison, the logConc was also included in the analysis, even though this was independent of the physico-chemical properties.

### 2.4. Applicability of the EFSA Pro Rata Approach

The EFSA Guidance on dermal absorption [3,8] recommends a pro rata correction for untested dilutions with lower active substance concentrations to address the concentration-related increase on dermal absorption. The pro rata approach uses a linear correlation of dermal absorption values as a conservative procedure based on a linear relationship of the lowest-tested concentration in terms of the highest dilution factor and the origin of coordinates. The linear relationship could then be extrapolated to an even lower and not tested concentration (i.e., higher dilution factor), using an experimental dermal absorption value of the same formulation/product and the respective dilution factor.

The dataset contains 130 studies, each including experiments with the concentrate and two dilutions, while, for three concentrates, three dilutions were tested. These data comprise several combinations of 89 different active substances in 66 different formulations and 60 active substances in solvent only. This sub dataset was evaluated regarding the applicability of the pro rata approach which applies to dilutions, i.e., to compare the extrapolated value of a second dilution based on the first dilution and to compare the extrapolated values with the experimental values of the second dilutions. An example of the pro rata calculation is provided in the EFSA Guidance on Dermal Absorption [3].

### 2.5. Cut-Off Criteria for Active Substance Concentration to Define Concentrates and Dilutions

For pesticides, default dermal absorption values can be used according to EFSA [3] where no specific data on the formulation in question are available. The EFSA Guidance on Dermal Absorption [3] set different default values for concentrated products and for their (in-use) dilutions. In contrast to the previous EFSA Guidance [8], thresholds were dismissed to distinguish concentrate products from dilutions (5% active substance concentration) due to the outcome of the analysis of data. No clear definition was provided to distinguish concentrates from dilutions. However, this categorisation was requested from many Member States to allocate the adequate and harmonised default value of dermal absorption. Especially for ready-to-use products, differing views were expected to result in different default values and non-harmonised assessments. Thus, the Standing Committee on Plants, Animals, Food and Feed (SCoPAFF) agreed on a pragmatic indication supplementary to EFSA [3], referring to the concentration threshold proposed by EFSA [8]: While a plant protection product is considered a “concentrate” when the active substance concentration is higher than 50 g/L (or 50 g/Kg or 5%), it is considered a “dilution” when the active substance concentration is lower than or equal to 50 g/L (or 50 g/Kg or 5%).

The default dermal absorption values recommended by EFSA [3] are as follows: (1) for organic solvent-formulated products or in other types of formulations, a default dermal absorption value of 25% may be applied for concentrated products, and a default dermal absorption value of 70% may be applied for (in-use) dilutions; (2) for water-based/dispersed or solid-formulated products, a default dermal absorption value of 10% may be applied for concentrated products, and a default dermal absorption value of 50% may be applied for (in-use) dilutions. It should be noted that these formulation categories were selected based on information on the chemical composition of the tested product, information on the phase in which the active substance is dissolved or emulsified/suspended and the expectable impact on dermal absorption [3]. The same categorisation has been used for comparison with the EFSA recommendation. The present dataset was analysed to estimate the impact (=underestimation of dermal absorption) of the set threshold of 5% active substance concentration to distinguish concentrate products from dilutions. For this, the dataset was subdivided into “organic solvent-based or other” and “water-based/dispersed or solid” based on the formulation categories according to EFSA [3].

## 3. Results and Discussion

### 3.1. Parameters and Characteristics of the Dataset

#### 3.1.1. Chemical Space of Substances and Distribution of Estimated Dermal Absorption

**Figure 2:**
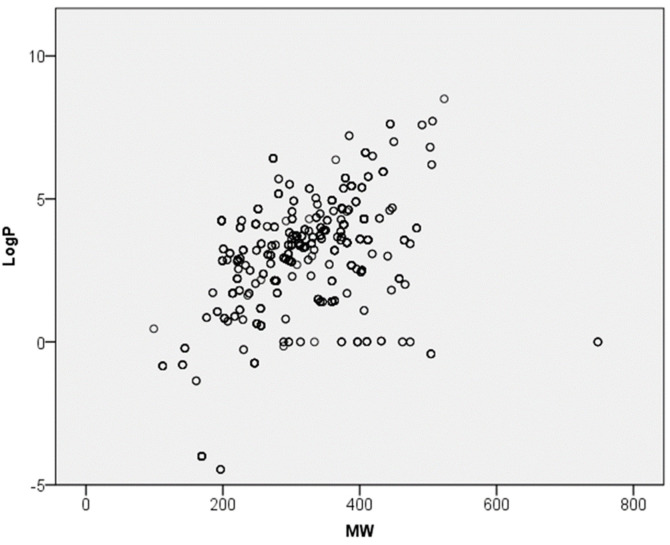
Chemical space of pesticide-active substances covered in the dataset. Chemical space is represented by molecule complexity as MW [g mol^−1^] LogP (n = 179 active substances).

The in vitro dataset comprises 179 pesticide-active substances with an MW range of 99.09 to 748.01 g mol^−1^, and a logP range of −4.46 to 8.5 (Figure 2). The active substances were tested in 353 different mixtures (189 with known detailed composition), resulting in a total of 945 experiments. Figure 2 demonstrates that the basic physico-chemical properties of the tested active substances are well-distributed.

The distribution for non-transformed endpoint percentage dermal absorption (sum of mean radioactivity in receptor fluid, skin and tape strips, refer to Section 2.1) indicated that the majority of the experiments resulted in lower percentage dermal absorption values. While 67.8% (n = 641) of the experiments exhibited a percentage dermal absorption below 10%, the remaining experiments were distributed with a decreasing frequency with regard to dermal absorption values. Only 0.7% of the experiments (n = 7) predicted a dermal absorption equal to or above 70%. As the percentage dermal absorption values do not fit to a normal distribution (Kolmogorov–Smirnov; Shapiro–Wilk, IBM SPSS Statistics, version 21), the data was log-transformed to achieve nearly normally distributed data.

#### 3.1.2. Mean Recovery/Mass Balance

Investigating the reference chemical testosterone, Heylings et al. [27] demonstrated that a mass balance of 99% could be achieved for testosterone using a radio-labelled substance and conducting the experiments according to a highly standardised protocol in agreement with OECD TG 428 [4]. While these results referred to one substance only (testosterone) and are based on an in-house database of in vitro dermal absorption studies on the pesticides of a single laboratory [28], Kluxen et al. [29] described the EFSA recovery criterion as not realistic based on the interpretation of statistical analysis of the EFSA dataset [3] on in vitro dermal absorption studies of pesticides, explaining these findings, e.g., with low laboratory standards. In the EU, EFSA requirements [3] have to be met for pesticides in dermal absorption studies performed according to OECD 428. A high level of recovery is required for pesticides, as this supports a low dermal absorption value. Otherwise, for risk assessment, missing material will be corrected via more conservative approaches [3]. Following this discussion, the mass balance distribution of the present dataset is evaluated to assess whether the mean recovery criterion of greater than or equal to 95%, as recommended by EFSA et al. [3], is realistic. Data with recovery rates greater than 105% usually result in an overestimation of dermal absorption and are not automatically rejected. They will generally be treated in the same way as data between 95 and 105%, and no distinction has been made in this evaluation.

**Figure 3:**
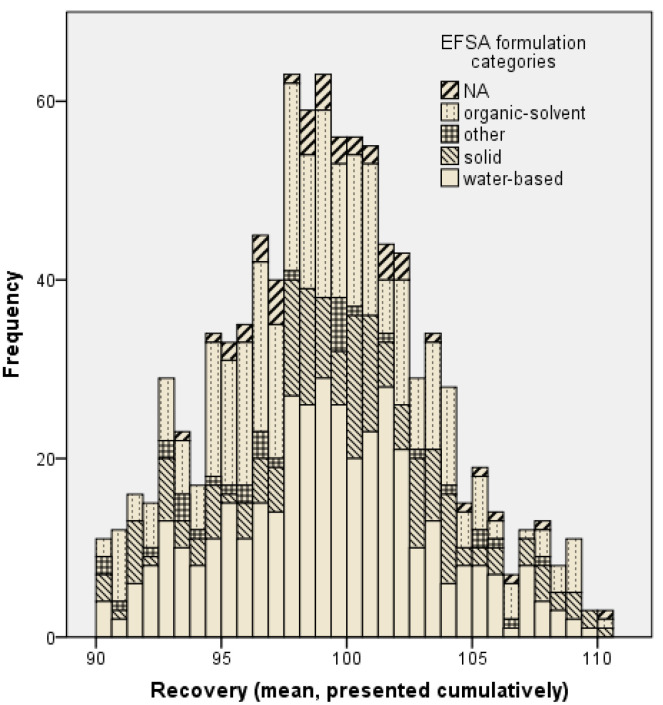
Histogram of mean recovery [%] for all experiments of dataset (n = 945 experiments). Mean recovery is shown cumulatively for the different formulation types of organic solvent-based, solid, water-based and other formulations, categorised according to EFSA (2017). NA = unknown formulation type. Mean recovery of all experiments presented as percentage of applied dose (radioactivity). Mean = 99.2, standard deviation = 4.21.

For reliability reasons, only studies fulfilling a mass balance of 90 to 110% of the applied dose, as recommended by the respective OECD documents [4,5,6], were recorded in the present dataset, as reported in Section 2.1. Referring to the presented dataset, the mean recovery of the various active substances for all experiments (n = 945) was 99.2%. The distribution of the respective mean recovery for all experiments is shown cumulatively for the different formulation categories according to EFSA et al. (2017) in Figure 3.

Table 1 presents several mass balance data from the dataset, i.e., 945 individual dermal absorption in vitro experiments (mean values of compartments over replicates) for 179 active ingredients of pesticides in 353 different mixtures, as described in Section 2.1. The presented dataset is differently split to investigate factors possibly influencing the mass balance. The OECD TG 428 was published in 2004 [4], but several guidance documents were made available in 2004 [5], 2011 [6] and 2012 [8] (revised in 2017, [3]) for the conduct and evaluation of dermal absorption studies on pesticides.

**Figure 4:**
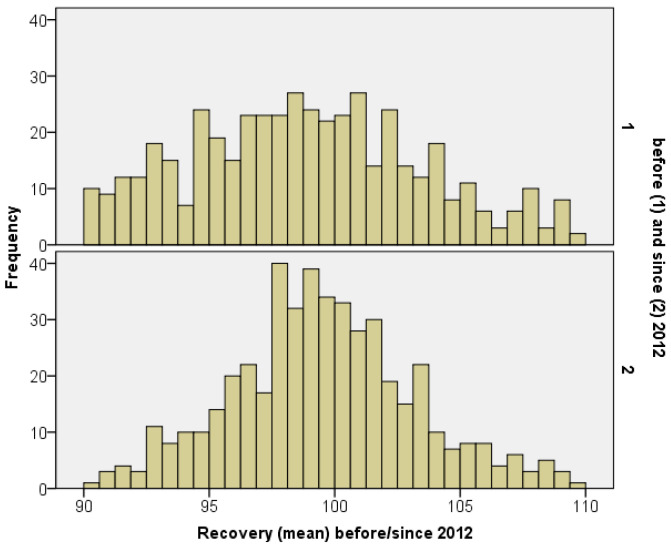
Frequency of mean recovery [%] for experiments of dataset received before and since the year of 2012. Mean recovery presented as percentage of applied dose (radioactivity). 1: experiments (n = 473) submitted before the year of 2012, mean = 98.9, standard deviation = 4.6. 2: experiments (n = 472) submitted since the year of 2012, mean 99.6, standard deviation = 3.7.

To investigate the possible impacts of published guidance documents for pesticides on the quality of data, the resulting mean recovery for experiments received before (n = 473) and since (n = 472) the year of 2012 are compared (see also Figure 4). It is shown that the mean recovery improved overtime from 98.9% with a standard deviation of 4.6% (studies from 1996 to 2011) to a mean recovery of 99.6% with a standard deviation of 3.7% (studies from 2012 to 2018), noting that the mean values are only of limited informative value. Obviously, the quality of data has been improved over time as the recovery of 89% of experiments conducted from 2012 to 2018 is above or equal to 95%, in contrast to 77% of experiments conducted from 1996 to 2011. Furthermore, there might be several influences on the recovery based on the difficulties of concentrates (e.g., technical handling) or of dilutions (e.g., limit of detection). Thus, data conducted from 1996 to 2018 are split—according to their dilution status—into concentrates and dilutions, but no clear difference is identified for the mean.

Additionally, lower recovery due to technical handling might be influenced by differences in formulation type, e.g., complicated dosing or washing and, thus, experiments are grouped based on the respective formulation type in analogy to formulation categories according to EFSA et al. [3] in water-based, organic solvent-based, solid and other formulations (Table 1). The means of the recovery of experiments with water-based and solid formulations are 99.2 and 99.8, respectively. For organic solvent formulations, the mean of recovery is 98.9 and, for other formulations, 97.9. Meanwhile, for water-based, organic solvent-based and solid formulations, more than 82% of the experiments meet the EFSA criterion, being greater than or equal to 95%, and only 68% of the experiments do the same for other formulations. Other formulations are a group of the following formulation types: bait concentrate (CB), capsule suspension (CS), gel for direct application (GEL/GD), bait, ready for use (RB), mixture of capsule suspension and suspension concentrate (ZC), seed coated with a pesticide (PS), experimental solution of active substances in solvent (AI).

Those formulation types might be somewhat “difficult-to-handle” formulations impeding correct dosing or recovering during the study conduct. Nevertheless, the contribution of these formulations to the experiments in the database is only 3.8% (n = 34) and plays a rather minor role. Almost 80% of the experiments were carried out with water-based formulations (43.1%) and organic solvent-based ones (33.6%). The distribution in the database allows some conclusions to be drawn about the distribution of registrations in Germany. Improvements in study quality might be due to a harmonised study conduct and the establishment of specific guidelines. It may also be due to the fact that analytical techniques and automated dosing have improved over time and laboratories have become more specialized and experienced with the method. In conclusion, considering the mean of recovery dermal absorption, and based on the number of dermal absorption studies achieving a mass balance greater than or equal to 95%, it is reasonable that this is an appropriate criterion. Moreover, it should be noted that data with lower recovery ranges are not rejected, but the missing material is addressed by the normalisation of data or addition of the missing amount. Since data with recoveries above 105% are not automatically rejected and treated in the same way as data ranging from 95 to 105%, no separate distinction was performed.

#### 3.1.3. Effect of Dermal Load and Formulation on Dermal Absorption

The highest mean percentage dermal absorption for human skin for concentrates using a concentration of 150 g/L was 39% for an active substance in a suspension concentrate, whereas the highest mean percentage dermal absorption for a diluted test formulation was determined to be 85% for another active substance, tested at a concentration of 3.95 g/L in an aqueous dilution of water-dispersible granules.

**Figure 5:**
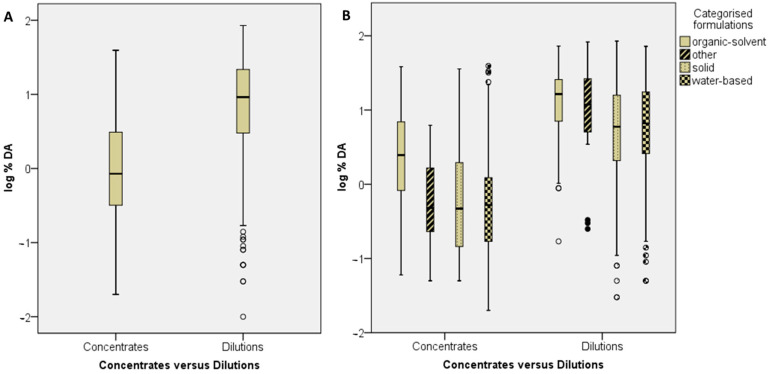
Boxplot of log%DA for concentrates (n = 368) and dilutions (n = 575). Outliers are indicated by circles and experiment number. n = 2 for not specified if concentrate or dilution were excluded from the dataset. (**A**) All the experiments of tested products are split based on the dilution status in concentrates and dilutions. (**B**) All the experiments of tested products are split based on the dilution status in concentrates and dilutions and separated into four formulation categories “organic solvent-based”, “water-based”, “solid” and “other” formulations according to EFSA (2017). n = 302 for organic solvent-based formulations (dispersible concentrate (DC): n = 2, emulsifiable concentrate (EC): n = 214, electrochargeable liquid (ED): n = 2, emulsion/oil in water (EW): n = 18, microemulsion (ME): n = 9, oil-based suspension concentrate (OD): n = 14, oil-miscible flowable concentrate (oil-miscible suspension) (OF): n = 4, SE: n = 39). n = 388 for water-based formulations (flowable (FL): n = 3, flowable concentrate for seed treatment (FS): n = 48, suspension concentrate (SC): n = 284, soluble concentrate (SL): n = 53). n = 176 for solid formulations (dustable powder (DP): n = 1, granule (GR): n = 1, water-soluble granules (SG): n = 8, water-soluble powder (SP): n = 4, water-dispersible granules (WG): n = 141, wettable powder (WP): n = 20, water-dispersible powder for slurry seed treatment (WS): n = 1); n = 34 for other formulations (experimental solution of active substance in solvent (AI): n = 10, capsule suspension (CS): n = 11, seed coated with a pesticide (PS): n = 1, bait/ready for use (RB): n = 6, mixed formulation of CS and SC (ZC): n = 6). n = 45 for NA (no information on formulation type) were excluded.

Figure 5A shows that, on average, the % dermal absorption for dilutions is significantly higher than the % dermal absorption for concentrates (Mann–Whitney U Test, *p*-value: 0.000, significance level 0.05: distribution and medians of log%DA is significantly different across the concentrate and dilution categories). Pesticide formulations are categorised based on their composition and the physico-chemical properties of the plant protection or biocidal product into one of the 65 formulation code groups specified by FAO/WHO [23]. A total number of 25 different formulation types are listed in the dataset. In the current dataset, many formulation types are not present in a sufficient number for the analysis. Thus, a grouping approach was followed to split the data into four formulation categories (organic solvent-based, water-based, solid and other formulations) based on the rationale for the grouping of formulation types given in EFSA et al. [3]. Figure 5B demonstrates the % dermal absorption for concentrates and dilutions, divided into the four formulation categories. Depending on the formulation category and the status of the dilution (concentrate/dilution), a ranking of the % dermal absorption could be made.

For concentrates, the highest median of log%DA is linked to organic solvent-based formulations, followed by water-based formulations, and finally, the other and solid formulation categories. For dilutions, the highest median of log%DA is linked again to organic-solvent based formulations, followed by other formulations, water-based formulations and solid formulations. For concentrates, the median log%DA is significantly higher for organic solvent-based formulations than for the remaining formulations (pairwise comparison, significance level 0.05), while the remaining formulations (water-based, solid, other) are not statistically significantly different.

For dilutions, the median log%DA of the organic-solvent and water-based formulation types are statistically significantly different; additionally, this applies to the solid and organic-solvent formulation types (pairwise comparison, significance level 0.001), while the remaining formulations (water-based vs. other, solid vs. other, solid vs. water-based, organic-solvent vs. other) are not statistically significantly different.

This result is in agreement with the general assumption that the organic solvent content in formulations strongly affects the dermal absorption of substances, e.g., by increasing the permeability of the skin or acting as carrier. Nevertheless, there are exceptions, and data must be carefully evaluated for individual cases.

It is also important to recognise that differences between formulations may be confounded by the properties of the active ingredient. This is because the type and composition of the formulation depends on the physico-chemistry of the active ingredient.

During risk assessment, data on dermal absorption are not always available for each product. In such a case, the possibility of transferring data from another tested formulation to the untested product must be considered. In particular, the various co-formulants contained and the concentration of the active substance are important impact parameters, which determine the extent of dermal absorption. Thus, the EFSA Guidance on Dermal Absorption [3] provides several conditions which should be met to assume that the tested and untested formulations are closely related, and the use of data from tested to untested formulations can be accepted. It is further stated that it is considered unlikely that the criteria on similarity will be met when moving from one formulation type to another (e.g., suspension concentrate to emulsifiable concentrate). Aggarwal [30,31] concluded, based on the analysis of data, that dermal absorption data generated with a solvent-based formulation could be used as conservative surrogates for primarily water-based/dispersed/suspended, and solid formulations. However, this statement is questionable, especially because of the doubtful grouping of the formulation types; the authors assumed OD (=oil dispersion) formulations to be water-based. According to FAO and WHO [23], OD formulations are suspensions of active ingredient(s) in an organic, non-aqueous fluid.

The analysis of the present data shows that this general assumption that the dermal absorption in organic solvent-based products can always be regarded as the worst case should be considered critically. Although the median log%DA of organic-solvent based concentrates is statistically significantly higher than medians of all the other concentrate formulations (water-based, solid, other), there is still a large overlap. Moreover, for dilutions, this overlap is even larger (see Figure 5). Therefore, the bridging of dermal absorption data between different formulation types requires case-by-case considerations. In particular, it should be kept in mind that even formulations belonging to the category “water-based” contain water as a major part, but a substantial content of organic solvents is not excluded here. The highest dermal absorption of a water-based concentrate in the dataset comes from an SC formulation. When looking at the exact composition (confidential information), it becomes apparent that a large proportion consists of water, but a smaller, still not negligible proportion of solvent is contained as well. This fact underlines that each formulation must be considered as a whole, in which every specific co-formulant, and the mixture of these, exert a respective influence on the dermal absorption of the active ingredient. The bridging of data in the sense of worst-case predictions to different products should, therefore, be considered with caution.

Moreover, it can be seen that for the dilutions of the different formulation categories, the difference in dermal absorption (median) becomes smaller (Figure 5B). Overall, this is conclusive, since the concentrates are often diluted with very large portions of water and, thus, the proportion of solvents and other relevant co-formulants (e.g., emulsifiers, wetting agents) is similarly lower across all the categories in dilutions. However, even at less than 1%, this type of co-formulant is still effective.

#### 3.1.4. Concentration Effect on Dermal Absorption of In-Use Dilutions

Generally, it can be assumed that the relative percentage dermal absorption is inversely related to the active substance concentration in most cases [26]. Buist et al. [26] explained this relationship with the saturation of the absorption capacity of the skin by the increasing dermal loading. When this capacity is saturated, the absorption rate will no longer proportionally increase with dermal loading, and, thus, the relative amount absorbed will decrease with increasing dermal loading. Especially for less water-soluble substances, the very slow rate of dissolution in the water film on the skin or its rapid saturation may be the cause of the levelling off of relative absorption in higher dermal loading ranges.

The present sub dataset with 147 experimental settings comprising a minimum of a tested concentrate and two tested dilutions was evaluated regarding the dependence of % dermal absorption on the area dose/the tested concentrations of the active substance (see Table S2). Since the area used within a study was identical for the three different concentrations, the dilution factors of the concentrations tested can be considered equivalent to the change in area dose in this analysis. In 99 of 118 experimental settings (=84% of data), there was an inversely proportional relationship between the tested concentration and relative dermal absorption of the active substance. However, in 19 out of 118 cases (=16%), this relationship was not observed. Two cases showed more or less constant percentage dermal absorption values over the tested concentrations/dermal loadings. For five cases, a proportional relationship between dermal loading and relative dermal absorption of the active substance was observed, meaning that the percentage dermal absorption values decreased with the dermal loading of the active substance. It was observed that there were also a few cases where there was no proportional relationship between dermal load and absorption. In 11 cases, the mean concentration was the highest; in two cases, the lowest. For the deviating cases, neither a specific formulation type nor any other plausible reason can be found, e.g., strong skin irritation of the concentrate vs. no skin-irritating property of the product as a reasonable explanation.

Within these data, the general assumption of the percentage dermal absorption of the active substance increase with lower concentration could be proved. For the small number of cases where this general assumption does not apply, further research is needed to improve the current pro rata correction approach. Currently, when extrapolating dermal absorption values to further dilutions, the inversely proportional behaviour should be confirmed with at least one dilution since this is a relevant aspect in the risk assessment for operators/workers/residents and bystanders exposed to pesticide (dried) dilutions.

#### 3.1.5. Experimental Parameters and Dermal Absorption

Besides the effect of formulation types on dermal absorption presented above, the impact of the experimental parameters, including skin type (dermatomed, full-thickness or isolated epidermis), occlusive or non-occlusive conditions, the cell systems (flow-through diffusion cells or the more classical static diffusion cells), and the exposure duration were compared to identify any possible influence on dermal absorption.

- Skin type

**Figure 6:**
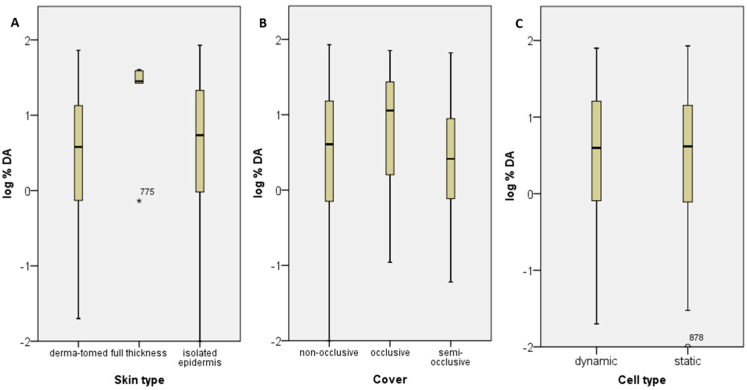
Boxplots of distribution of log%DA relating to several experimental parameters. (**A**) Comparison of log%DA with regard to the used skin type (dermatomed, full-thickness or isolated epidermis). Dermatomed skin: n = 691, full-thickness skin: n = 5, isolated epidermis: n = 221, experiments with no information on the skin type used (NA, n = 6) or not-specified skin (scissors, n = 22) were excluded. Outlier marked with an asterisk. (**B**) Comparison of log%DA with regard to the state of occlusion (=cover, i.e., non-occlusive, occlusive or semi-occlusive). Non-occlusive: n = 579, occlusive: n = 53, semi-occlusive: n = 114, experiments with no information on the state of occlusion were excluded (NA, n = 199). (**C**) Comparison of log%DA with regard to the used cell system under flow-through (dynamic, n = 426) or static (n = 519) conditions.

In Figure 6A, the distribution of % dermal absorption relating to the used skin type is presented. Altogether, three different skin types were used in the experiments available in the dataset: dermatomed, full-thickness or isolated epidermis. The distribution of log%DA is the same across the two categories of skin type, dermatomed and isolated epidermis, as evidenced by independent-samples Kruskal–Wallis test (*p*-value: 0.275, significance level 0.5). The skin type “full-thickness” is excluded from the analysis because only five experiments were conducted using full-thickness skin; however, this group is statistically significant different (Kruskal–Wallis test, *p*-value: 0.000, significance level 0.05). In EFSA et al. [3], a certain thickness of the skin membrane is already recommended in order to achieve comparable data.

- State of occlusion

Figure 6B presents the % dermal absorption distribution for experiments using non-occlusive, occlusive or semi-occlusive conditions. The evaluated in vitro dermal absorption experiments on human skin show a statistically significant influence of the state of occlusion on % dermal absorption (borderline, Kruskal–Wallis test, *p*-value: 0.005, significance level 0.05). The comparison shows that a higher median of % dermal absorption might be achieved under occlusive conditions. This result may be reasonable because, in occlusive conditions, the skin could remain more moistened and, therefore, more permeable to certain substances. However, this analysis might be confounded by the physico-chemical properties of the active substance present in the sub-sets and a comparative study may be needed to corroborate this finding. According to OECD TG 428, “Normally, donor chambers are left unoccluded during exposure to a finite dose of a test preparation. However, for infinite applications and certain scenarios for finite doses, the donor chambers may be occluded”. These data provide evidence for the fact that occlusion influences certain parameters that may affect skin permeation, as already described by Hopf et al. [32]. It is reasonable that it may be necessary to work under occlusion in some cases; for example, when the test substance is volatile, or to mimic a scenario where the skin is contaminated and covered, e.g., with protective gloves. Possibly, respective guidelines should provide more guidance on the scenario to be selected.

- Cell type

The in vitro approach uses either flow-through diffusion cells or the more classical static diffusion cells. While flow-through diffusion mostly requires dosing from a pipette because cells are fixed in a heated block apparatus, the dose can be applied to the skin surface by mass using static diffusion cells. Figure 6C compares the distribution of % dermal absorption when flow-through diffusion cells, or the more classical static diffusion cells, are used. The medians of log%DA are not significantly different across the categories of cell type (*p*-value of 0.080 for independent sample median test, significance level 0.05). No relevant influence on dermal absorption is identified for the cell system, confirming the results already published [33,34]. The advantages of the flow-through diffusion cells are mainly in handling and automatic sampling. However, the influencing variables are difficult to identify here, since the database is very inhomogeneous in the selection of experimental parameters, such as the size of the skin membrane, flow rate, receptor fluid, etc.

- Relationship between cell type and surface area

**Figure 7:**
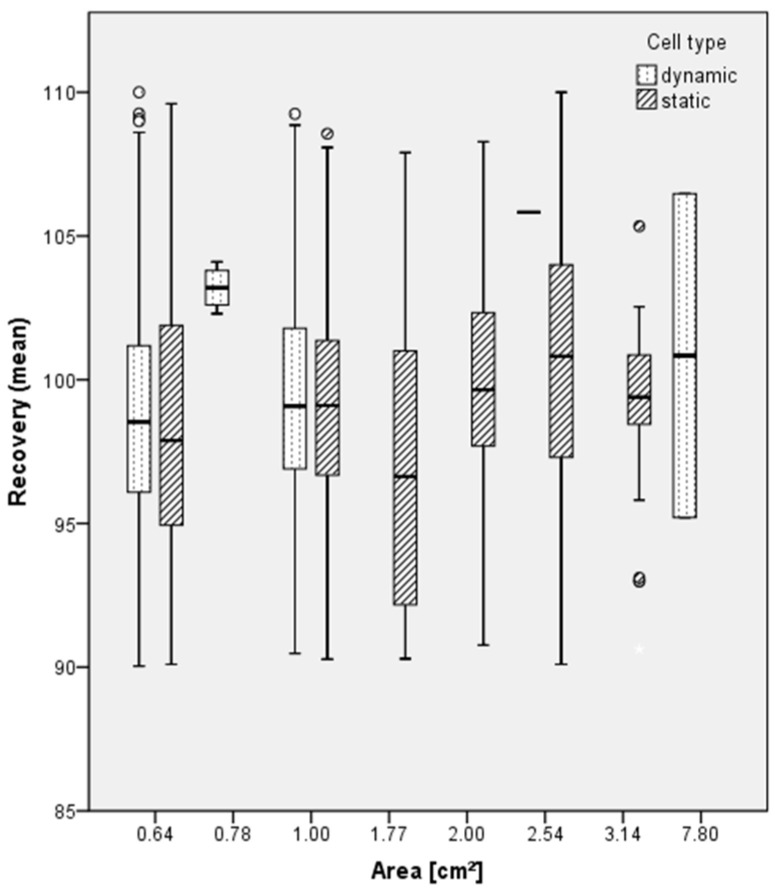
Mass balance [%] of in vitro dermal absorption studies on the pesticides available at the BfR, differently grouped based on the used cell system (dynamic (flow-through) or static diffusion cell) and the used area of skin in cm^2^. Dynamic cells: n = 319 for 0.63 and 0.64 cm^2^ (combined), n = 4 for 0.78 cm^2^, n = 96 for 1 cm^2^, n = 1 for 2.54 cm^2^, n = 2 for 7.8 cm^2^, n = 4 for not specified were excluded from the dataset; Static cells: n = 60 for 0.6 and 0.64 cm^2^ (combined), n = 110 for 0.95 and 1 cm^2^ (combined), n = 44 for 1.77 cm^2^, n = 39 for 2 cm^2^, n = 193 for 2.53 and 2.54 cm^2^ (combined), n = 63 for 3.14 cm^2^.

To investigate whether there is a relationship between the surface area of the used skin and performance in terms of recovery due to technical dosing possibilities, Figure 7 demonstrates the mean recovery versus different skin areas used in the two cell systems (static/dynamic).

Comparing the skin area of 0.64, 1.0, 1.77, 2.00, 2.54 and 3.14 cm^2^ of static and dynamic diffusion cells, the median of the recovery is quite similar and a significant difference is not identified. Obviously, a clear difference in recovery cannot be identified. Performance in terms of recovery does not depend on the used area of the skin membrane in static or dynamic cell types. The same applies for the cell system, as, for example, there is no difference in recovery when data on flow-through diffusion cells are compared to static cell using a skin surface area of 1.0 cm^2^. In summary, the size of the cells/surface area does not seem to affect the mass balance in a relevant way, but is mainly driven by considerations that accommodate the diffusion cell operation and analytical requirements.

- Exposure duration

According to OECD TG 428, “skin exposure to the test preparation may be for the entire duration of the experiment or for shorter times (i.e., to mimic a specific type of human exposure)”. EFSA [3] specifies that exposure should mimic a working day and should, therefore, be between 6 and 10 h, with a sampling period of at least 24 h. However, studies sometimes use longer exposure durations, e.g., 24 h. It can be assumed that, especially for substances with slow absorption, the duration of exposure is a relevant factor for the total absorption.

**Figure 8:**
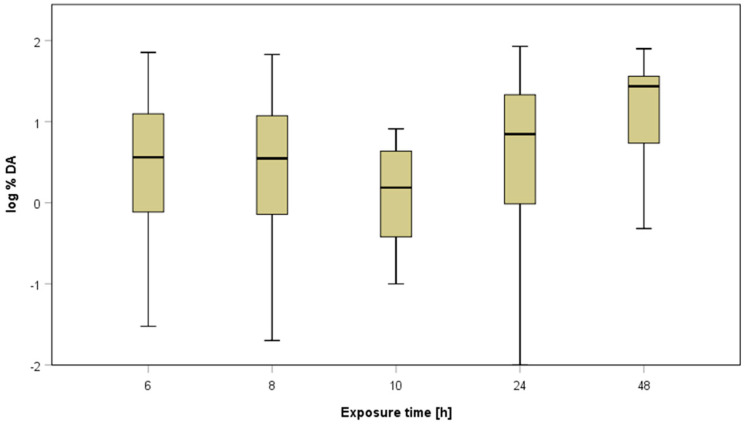
Boxplots of distribution of log%DA relating to several exposure times (i.e., application time of dose on skin membrane). Comparison of log%DA with regard to the time (6, 8, 10, 24 or 48 h). 6 h: n = 297, 8 h: n = 414, 10 h: n = 6, 24 h: n = 189, 48 h: n = 35, experiments with no information on exposure time (NA, n = 2) or 7 (n = 1) and 9 h (n = 1) were excluded.

The present dataset of 941 experiments contains data with an exposure duration of 6 to 10 h, but also some experiments with an exposure duration up to 24 or 48 h. About 76% of the experiments are available using an exposure duration of 6 or 8 h (in total, 711 experiments), reflecting a working day. Less data are available using an extended exposure duration of 24 h (n = 189), or even 48 h (n = 35). Comparing the mean dermal absorption values of all available data (see Figure 8), no difference in the distribution of dermal absorption values can be demonstrated with exposure durations of 6, 8, 10 or 24 h. For 48 h exposure duration, higher values were achieved; however, especially 48 h exposure duration might represent a critical time with respect to skin membrane integrity. Because different exposure durations were used for different test formulations, a conclusion from the whole dataset about the effects of exposure duration should be taken with caution. Therefore, a sub dataset of formulations was created, for which studies with different exposure durations are available in order to investigate more precisely the contribution of exposure time to dermal absorption.

**Figure 9:**
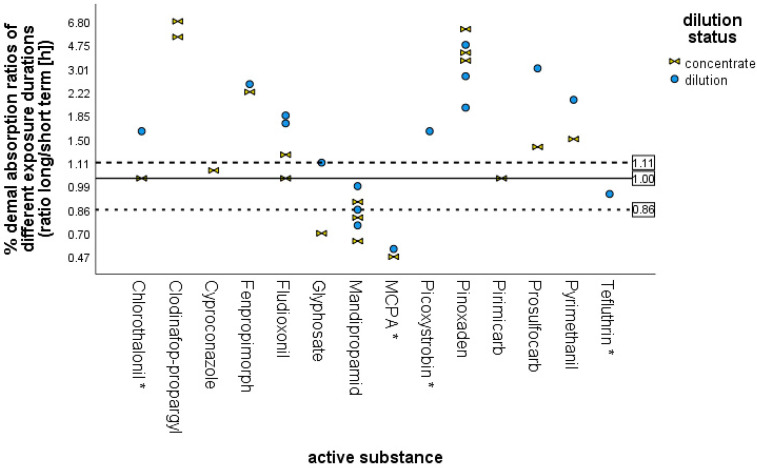
Scatterplot of dermal absorption ratios of different exposure durations obtained in 34 experiments for 14 different active substances. Each point corresponds to the % dermal absorption ratio of one formulation (concentrate or dilution) of the same active substance (same formulation) tested with an exposure duration of 24 h (long-term) and 6, 8 or 10 h (short-term). The horizontal (x-) axis shows different active substances for which data are available with different exposure durations for the same formulation. The vertical (y-) axis shows the ratio of the mean percentage dermal absorption for an exposure duration of 24 h to the mean percentage dermal absorption for a shorter exposure duration (6, 8 or 10 h), either for a concentrate, or for a dilution. n = 34. Reference continuous line for y (ratio of % dermal absorption values for different exposure durations) = 1. Upper dotted line at a ratio of % dermal absorption values for different exposure durations of 1.11, lower dotted line at a ratio of 0.86. Substances marked with an asterisk (MCPA, tefluthrin, chlorothalonil and picoxystrobin): sampling was continued until 24 h in total, even if the exposure was for a shorter period of time (e.g., exposure duration 6 h, but sampling time up to 24 h total). For the other active substances, sampling was not continued after the exposure was terminated, e.g., exposure duration 6 h, then sampling time was also 6 h.

The dataset includes studies for 14 active substances, each in one formulation but with two different exposure times: long (24 h) and short (6, 8, or 10 h). For 10 substances, both the concentrate and a dilution were tested with two exposure durations; for three substances, only the concentrate or a dilution was tested with two exposure durations. In order to further investigate an influence of the exposure time, the ratios of the mean dermal absorption values of a formulation (concentrate or dilution) of the same active substance (same formulation) tested with an exposure duration of 24 h (long-term) and 6, 8 or 10 h (short-term) were calculated (Figure 9). Ratios smaller than 1 mean that dermal absorption was smaller at an exposure of 24 h than at shorter exposure durations, whereas ratios above 1 mean that dermal absorption was greater for an exposure duration of 24 h than for shorter exposure durations.

Figure 9 shows that 9 out of 34 experiments have a ratio in the range of 0.86 to 1.1; Here, the different exposure duration has no relevant influence on dermal absorption. Figure 9 demonstrates that for 19 out of 34 experiments, however, the mean dermal absorption value is higher when an exposure duration of 24 h was used, irrespective of dilution status (concentrate or dilution). This result may not be surprising, especially for substances that are absorbed more slowly, when considering that studies with shorter exposure time are usually terminated at 24 h or earlier. The data also show, on the other hand, that, in 6 experiments, the ratio is in the range of 0.47 to 0.78, meaning that the dermal absorption is higher in studies after a short exposure than in studies after a long exposure time of 24 h, which does not seem scientifically justifiable. The intra- or inter-variability of skin samples might be one explanation for this result. It should be noted that only for the active substances MCPA, tefluthrin, chlorothalonil and picoxystrobin, samples were taken up to 24 h for both exposure durations (e.g., exposure duration was 6 h, but sampling time was up to 24 h in total). For the other active substances, sampling time was analogous to the exposure duration (e.g., when exposure duration was 6 h, the sampling time was also 6 h). Thus, in the end, for the active substances MCPA, tefluthrin, chlorothalonil and picoxystrobin, the difference in dermal absorption ratios based on exposure duration was small, i.e., the exposure duration was of minor importance with respect to the influence on dermal absorption. The results for the 4 substances, for which the sampling was continued until 24 h in total, even if the exposure was for a shorter period of time, show that the permeation of the main part of the substance occurs within the first 6 h, either into the receptor fluid, or at least into the skin membrane, forming a depot which can be absorbed more or less even after termination of exposure by washing procedure.

The other substances (except mandipropamid and glyphosate) demonstrate an impact of exposure duration and sampling time on dermal absorption. As noted above, for these substances, sampling was not continued after the exposure was terminated, and, mostly, the mean dermal absorption values were higher when an exposure duration of 24 h was used. Further, compared with the results of the experiments of active substances with a sampling period up to 24 h, regardless of the exposure duration, permeation of the main part of the substance seems to occur within the first 6 h into the skin membrane, forming a depot. Considering the dilution status tested (i.e., concentrate or dilution irrespective of the dilution rate), it seems that the difference in dermal load may not be the main impact factor on the dermal absorption ratios of different exposure durations; for some substances, results are in the same range (see Figure 9).

Overall, based on the sub dataset, it can be concluded that the permeation of the main part of the substance usually occurs within the first 6 h, either into the receptor fluid, or at least into the skin membrane, forming a depot which can be absorbed more or less even after the termination of exposure by washing procedure. The exposure duration was of minor importance with respect to the influence on dermal absorption. The exposure time frame of 6 to 10 h, with an extended sampling time of 24 h, is in agreement with the risk characterisation because the toxicological reference values are based on daily doses.

#### 3.1.6. Substance Parameters and Possible Relation to Dermal Absorption

For the identification of a possible relationship between the dermal absorption and selected physico-chemical parameters of the active substances contained in the dataset, a variety of criteria were investigated. The analysed physico-chemical parameters for the active substances were the molecular weight, log octanol–water partitioning coefficient (logKow), molecular refractivity, Hbond acceptor, Hbond donor, topological polar surface area (TpSA), Van der Waals surface area, the logarithm (base 10) of the distribution coefficient (logD), molecular polarizability, receptor medium and the logarithm (base 10) of the concentration (logConc).

- Correlation coefficient

The correlation coefficient was determined as a measure of the degree of linear correlation between one of the selected parameters of the substance and dermal absorption; here, log%DA (see Table 2). Values closer to zero suggest that there is lower linear correlation between the parameters and log%DA. Taking into account the calculated correlation coefficients as well as the respective *p*-value, which indicates whether or not the predictor is meaningful for the model, parameters suggesting a better correlation to the dermal absorption are logKow, Hbond donor, Hbond acceptor, Van der Waals surface area, molecular polarizability, and logConc.

- Akaike Information Criterion

Specific active substance-related variables were selected in a stepwise approach to identify Akaike Information Criterion (AIC), which is an estimator of prediction error and the relative quality of statistical models for a dataset. A lower AIC value indicates a better-fit model. Table 3 shows that the chosen parameters logK_ow_, Hbond acceptor count, Hbond donor count, V-d-W surface area, molecular polarizability and logConc give the theoretically best model for predicting dermal absorption (model no 6).

- Stepwise regression

Stepwise regression was conducted with the parameters obtained in model number 6. For the regression, the degrees of freedom, the sum of squares, the residual sum of squares (RSS) and the AIC were used. RSS measures the level of variance in the error term, or residuals, of a regression model. The smaller the residual sum of squares, the better your model fits the data, and a value of zero means the model is a perfect fit. The same applies to the sum of the squares.

Table 4 presents the calculated quality criteria. LogK_ow_ is the parameter with the best fit as it shows the lowest values for the sum of squares, RSS and AIC. Interestingly, the logK_ow_ parameter did not show the highest correlation to the log%DA in the single parameter analysis, as presented in Table 2. Parameters with statistical significance do show a generally good fit in the regression (Hbond acceptor, donor, V-d-W surface area, molecular polarizability). However, when considering the variable logConc, it becomes apparent that even though the strongest linear relationship is shown (see also Table 2), this variable is the least suitable for determining dermal absorption when considering AIC, as provided in Table 4 (model fit with a higher AIC less suitable). This seems to be in contradiction with the assumption that the concentration has a major influence on the relative absorption through the skin. These results may show the difficulties in the development of the prediction models of dermal absorption values by the use of the parameters of the active substance. As known, the composition of the formulation strongly affects dermal absorption of the active substance. The results may indicate that information on the components of the mixture may help to improve the in silico methods of dermal absorption value prediction.

### 3.2. Applicability of the EFSA Pro Rata Approach

Especially for the application of pesticides, the product label often recommends several in-use dilutions of a concentrate depending on the crops to be treated. Also, for biocidal products, different in-use dilutions might be relevant to treat target organisms. However, dermal absorption studies performed with the product and one or more dilutions sometimes do not cover the relevant concentration recommended on the label. For untested dilutions with lower active substance concentration, the EFSA Guidance on Dermal Absorption [3,8] recommends a correction to address the concentration-related increase in dermal absorption with a decrease in dermal loading (=pro rata correction). According to EFSA [3], the pro rata correction is considered to be a conservative but appropriate approach in the absence of data and is a concept of worst-case linear extrapolation. The proposed application of the pro rata correction up to a maximum of a 5-fold difference and a cut-off at 30% dermal absorption [30,31] was not supported by the analysis of the EFSA dataset of in vitro dermal absorption studies on human skin [3].

**Figure 10:**
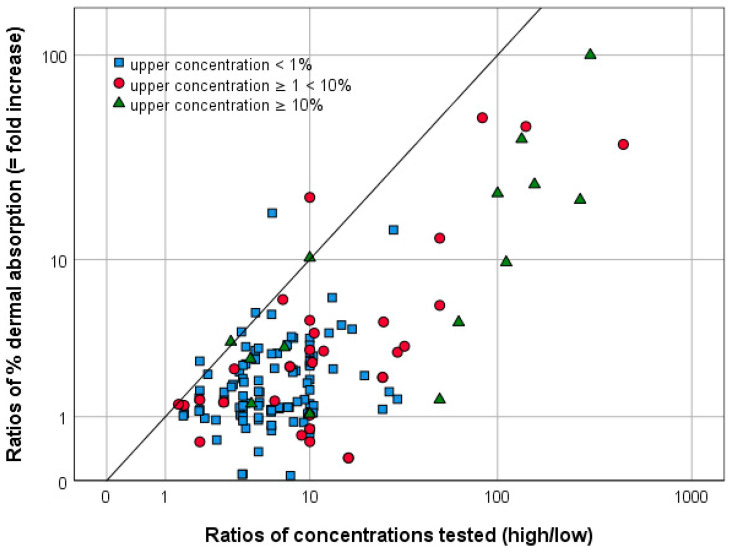
Scatter plot of the applicability of pro rata extrapolation for dilutions. Each point corresponds to the concentration ratio versus the % dermal absorption ratio of two dilutions of the same active substance (same formulation). The horizontal (x-) axis shows the ratio of the concentrations for the two dilutions. The vertical (y-) axis shows the ratio of the mean percentage dermal absorption for the lower concentration to the fraction mean percentage dermal absorption at the higher concentration. n = 147; logarithmic scale for both axes. Reference line for y (experimental % dermal absorption values) = x (extrapolated % dermal absorption values). Cases where (a) the upper concentration is <1% are shown in blue (square), (b) the upper concentration is ≥1% <10% are shown in red (circle), and (c) the upper concentration is ≥10% are shown in green (triangle).

The present dataset contains 120 human in vitro experimental settings that have tested dermal absorption of the same active substance for multiple concentrations (concentrate and two or more dilutions). In two of the experimental settings, different parameters, i.e., exposure time (10 h and 24 h) and skin (isolated epidermis and full-thickness skin) were tested but were, therefore, excluded from the analysis. For experimental settings with more than two tested dilutions, the closest dilutions were compared with each other, resulting in 147 experimental settings (in this case, 147 pairs of two aqueous dilutions, each of the same formulation with the same active substance) being suitable for the investigation of whether the pro rata approach might be overly conservative, or more or less appropriate for default assumptions. Figure 10 shows the comparison of concentration ratios and the ratios of % dermal absorption of the respective data. Seven out of 147 (5%) data points are above the linear line, highlighting the EFSA pro rata extrapolation, which means that the experimental mean % dermal absorption increased by a factor that was higher than the quotient of the concentrations tested. In these cases, the extrapolated % dermal absorption is an underestimate of the experimentally determined value. Underestimation of dermal absorption would be critical for pesticide risk assessment; although, it should be noted that an overestimation could also make informed risk management decisions difficult. The dates of conduct of the seven cases were 1996, 2001, 2009, two times 2012, 2013 and 2014. Thus, these higher values do not come from older studies only. Taking a closer look at these seven cases, it becomes clear that, for five of them, the ratio between the increase in mean % dermal absorption and decrease in the concentration tested was between 1.02 and 1.33. Due to the variability of the test method, this level of deviation can be considered acceptable and does not invalidate the pro rata approach. However, for the remaining two cases, the mean % dermal absorption increase was 2.06 and 2.84 greater than that extrapolated using the pro rata approach. Based on the available dataset, this means that 1.4% are not covered by the pro rata approach.

Figure 10 illustrates the relationship between the concentration range used for extrapolation and the fold increase in dermal absorption (ratio of % dermal absorption). Even though no clear difference can be seen, Figure 10 shows that, with increasing concentration, especially from concentrations higher than 10%, the extrapolation method might result in more overestimated values.

Referring to the formulation categories introduced by EFSA [3] (refer to section above), 89 of the 147 data points are dilutions of water-based or solid formulations. For 26 cases (29%), the theoretically extrapolated values are above 50%, which means that the default value of 50% dermal absorption would be used for risk characterisation instead of the extrapolated value. A total of 49 out of 147 data points are dilutions of organic solvent-based or other formulations. For 25 (51%), the theoretically extrapolated values are above 70%, which means that the default value of 70% dermal absorption would be used for risk characterisation. Nine of the 147 data points represent an active substance in solvent not suitable for categorization according to EFSA [3], and, thus, were not considered in detail here. The crop protection industry repeatedly objected that the pro rata approach is overly conservative. Aggarwal et al. [30,31] proposed the application of the pro rata correction up to a maximum of a 5-fold difference and a cut-off at 30% dermal absorption.

The analysis of the dataset gave the following results when considering the pro rata approach. On the one hand, 1.4% of the cases were not covered by the pro rata approach. On the other hand, with increasing concentration rates, especially from concentrations greater than 10%, the extrapolation method frequently leads to overestimates. Further, for one-third of the dilutions of water-based formulations, and half of the dilutions of organic solvent-based formulations, the pro rata extrapolation would result in unrealistically high values for dermal absorption.

It is noted that sampling at highly restricted doses may be somewhat inappropriate due to depletion, whereas dermal toxicity testing at high doses may show dose dependence due to saturation, as mentioned by Kissel [25]. The percentage dermal absorption is somewhat dependent on mass loading on skin. It is, therefore, important to standardise the amount applied for the in vitro study according to the recommendations in order for it to be comparable with the relevant exposure conditions of operators, workers and consumers. Nevertheless, the presented analysis shows that the current EFSA pro rata extrapolation is a useful tool to reduce the need for further studies in risk assessment, as mostly all cases were covered. Thus, the extrapolation approach is considered applicable for the regulatory assessment of pesticides. However, this data also show that there is still some potential for further improvement considering the level of overestimation.

### 3.3. Cut-Off Criteria for Active Substance Concentration to Define Concentrates and Dilutions

According to EFSA [3], a default dermal absorption value of 25% and 70% may be applied for concentrated products and for (in-use) dilutions for organic solvent-formulated products or in other types of formulations in the absence of product-specific information on dermal absorption. For water-based/dispersed or solid-formulated products, a default dermal absorption value of 10% and 50% may be applied for concentrated products and for (in-use) dilutions, respectively. The EFSA default dermal absorption values are based on a large dataset on pesticides and take into account the 95% upper confidence/credibility limit of the 95th percentile of the respective empirical relative absorption data for the different formulations. In order to achieve a harmonized approach to differentiate between concentrate and dilution, additional rules using a concentration limit at 50 g/L (or g/kg) of active substance content were agreed as feasible [35].

**Figure 11:**
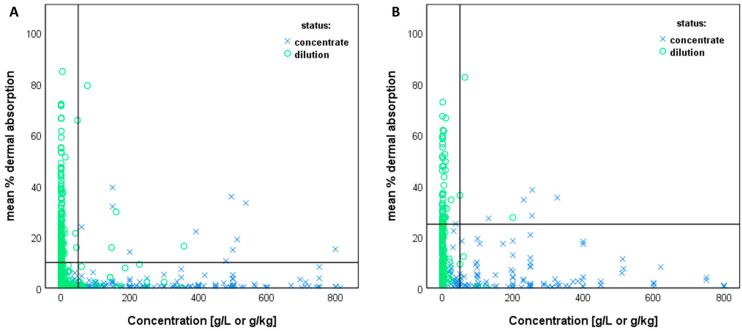
Scatter plots of the dermal absorption values [%] of different formulation categories versus the active substance concentration [g/L or g/kg]. The categorisation of concentrate products and dilutions was performed according to EFSA (2017). (**A**): Water-based and solid formulations, n = 564. Concentrate products are shown in blue (cross), dilutions are shown in green (circle). The horizontal line corresponds to the 10% default value for the respective concentrates. The vertical line corresponds to the set limit for active substance content (50 g/L or g/kg). (**B**): Organic solvent-based and other formulations, n = 336. Concentrate products are shown in blue (cross), dilutions are shown in green (circle). The horizontal line corresponds to the 25% default value for the respective concentrates. The vertical line corresponds to the set limit for active substance content (50 g/L or g/kg).

For the regulatory risk assessment of pesticides, only the underestimation of dermal absorption is relevant, as this may represent a risk to human health. Thus, the present data were analysed for the underestimation of dermal absorption when default values are used. In the case that the experimental value exceeds the default value, the dermal absorption is considered to be underestimated. The dataset presented here consists of 945 individual dermal absorption in vitro experiments (mean values of compartments over replicates) for 179 active ingredients of pesticides in 353 different mixtures (=formulations). In total, 45 of the data have no specified formulation type (e.g., active substance in solvent only, or formulation type not recorded in the dataset) and were excluded from evaluation, resulting in 900 data points of in vitro experiments. Figure 11 shows the distribution of the mean percentage values of water-based and solid formulations (Figure 11A), and organic solvent-based and other formulations (Figure 11B) with respect to the active substance concentration [g/L or g/kg].

For the analysis of the impact of the set concentration threshold (50 g/L or g/kg active substance content) on dermal absorption (underestimation), all products with active substance content below or equal to 50 g/L or g/kg are considered as dilutions (left side of the vertical line). The default value for dilutions would be applied in the absence of product-specific data. All products/test concentrations with an active substance content above 50 g/L or g/kg are considered as concentrates (right side of the vertical line). Notably, some underestimation is accepted for dilutions as well as for concentrates, because the default values recommended by EFSA [3] do not cover all individual cases, but, for derivation, the 95% upper confidence/credibility limit of the 95th percentile of the respective empirical relative absorption data was taken into account. The present analysis focuses on the additional underestimation of dermal absorption that occurs due to the application of the concentration threshold of 50 g/L or g/kg. In this concentration range, underestimation occurs in those cases when the experimental percentage dermal absorption values of dilutions are above the respective default dermal absorption values used for concentrates (10% or 25%, right side of the vertical line and above the horizontal line, refer to Figure 11A,B).

Referring to the present data, 0.7% (4 of 564 experiments) of the tested dilutions of water-based and solid formulations would be underestimated using the concentration threshold of 50 g/L or g/kg, i.e., they have a higher experimentally determined mean percentage dermal absorption value than the assigned default value for concentrates (10%). For dilutions of organic solvent-based and other formulations, 0.6% of all the tested formulations (2 of 336 experiments) would be underestimated using the concentration threshold of 50 g/L or g/kg (meaning that the experimentally determined mean percentage dermal absorption value is higher than the default value for concentrates (25%)).

The additional underestimation due to the use of the concentration threshold of 50 g/L or g/kg for the distinction between concentrate and dilution is low and of less relevance for risk assessment. These data show that setting up additional rules using a concentration limit at 50 g/L (or g/kg) active substance content is a pragmatic approach acceptable for harmonisation.

## 4. Conclusions

The BfR database dermal absorption presents a large dataset on measured dermal absorption values from studies submitted for the authorisation of plant protection products and biocidal products in Germany, or active substance evaluation in the EU. The data were collected in a systematic way to allow for an evaluation of selected parameters influencing dermal absorption.

The BfR database on dermal absorption provides a powerful tool with the collection of dermal absorption data for risk assessment. Within this large dataset, current approaches to dermal absorption can be evaluated and further aspects can be investigated.

Possibly due to the implementation of specific guidelines, e.g., EFSA [3,8] and OECD [6], study performance improved over time, as seen for the parameters‘ mass balance.

Further analyses could not identify a clear dependence of dermal absorption on some experimental parameters. Nevertheless, as already discussed by Hopf et al. [32], there is still a lack of precise guidelines for some experimental parameters during study conduct, such as cell parameters, receptor fluid, sampling, skin preparation; thus, a revision of guidelines such as OECD TG 428 could improve the reliability of test results. Acceptance criteria for the measurements of skin barrier function, some of which are defined in the literature (e.g., electrical resistance measurements [36]), are also not provided in the available guidance documents. These should also be considered as a critical quality criterion for which data should be provided by laboratories.

Approaches such as the pro rata correction for untested dilution rates, default values in the absence of data for different formulation categories, or the mass balance criteria recommended by EFSA [3] were supported by the present analysis.

An investigation was conducted to determine a possible correlation between dermal absorption and certain physico-chemical properties of the active substances, as well as their concentrations or the receptor fluids used. An additional analysis of the specific co-formulants is required to identify influencing factors that may be of greater importance than the experimental variables. In a recent publication, a predictive relationship between the available parameters (physicochemical properties, formulation type categories, concentration tested) from in vitro studies and the potential dermal absorption using a random forest model was discussed [37]. However, the present BfR database provides further information on the individual co-formulants and, thus, might offer a possibility for further analysis.

## Figures and Tables

**Table 1:** Mass balance [%] of in vitro dermal absorption studies on pesticides available at the BfR. Data is differently grouped based on the year of conduct (before or since published guidance documents), dilution status (concentrate (i.e., undiluted product) or dilution (i.e., dilution of the product)) and formulation category according to EFSA et al. (2017, [3]) (water-based, organic solvent-based, solid or other formulations).

Year	1996 to 2018	1996 to 2011	2012 to 2018	1996 to 2018	1996 to 2018	1996 to 2018	1996 to 2018	1996 to 2018	1996 to 2018
Dilution Status				Concen-trates	Dilutions				
Formulation Category						Water Based	Organic-Solvent Based	Solid	Other
Mean [%]	99.2	98.9	99.6	99.5	99.1	99.24	98.97	99.77	97.89
SD	4.21	4.6	3.7	4.1	4.3	4.02	4.2	4.6	5.0
n	945	473	472	368	575	388	302	176	34
Min [%]	90.04	90.04	90.57	90.1	90.04	90.04	90.21	90.1	90.28
Max [%]	110.0	110.0	110.0	110	109.6	109.57	110	110	108
≥95%	788 (83%)	366 (77%)	422 (89%)	319 (87%)	468 (81%)	326 (84%)	251 (83%)	145 (82%)	23 (68%)
≥95 ≤105%	702 (74%)	318 (67%)	384 (81%)	284 (77%)	418 (73%)	293 (76%)	227 (75%)	126 (72%)	18 (53%)

**Table 2:** Correlation and linear regression analysis of log%DA with physico-chemical parameters and log-transformed concentration of the tested active substance of the complete dataset. Significance codes: <0.001: ***; >0.001 <0.01: ** (not observed for the results); >0.01 <0.05: *; >0.05 <0.1: +.

Parameter	Correlation Coefficient	Linear Model Estimate	t-Value	*p*-Value	Statistical Significance
molecular weight	0.0222454	−0.0001709	−0.249	0.803370	
LogK_OW_	0.1317092	0.0304536	1.770	0.076992	+
molar refractivity	0.0514431	−0.0010800	−0.269	0.787763	
Hbond acceptor	−0.1205347	−0.1137090	−3.702	0.000227	***
Hbond donor	−0.1112570	−0.0734481	−2.291	0.022177	*
TpSA	−0.1153558	0.0022907	1.116	0.264688	
V-d-W surface area	0.0687760	0.0031440	5.060	5.05 × 10^−7^	***
LogD	0.1061477	−0.0099494	−0.492	0.622536	
molecular polarizability	0.0384758	−0.0423678	−3.471	0.000541	***
receptor medium	0.0015835	−0.0011920	−0.061	0.950988	
LogConc	−0.5318026	−0.3369989	−20.491	<2 × 10^−16^	***

**Table 3:** Variable selection with stepwise approach to identify dependence on log%DA. Based on the full linear model (1), further parameters were removed stepwise to enhance the resulting AIC values until all the remaining parameters were significant (specified cut-off 0.1) using the stepwise regression with R software (R Core Team 2020; RStudio, version 1.3.1093, © 2009–2020 RStudio, PBC). The selected variables were the physico-chemical parameters of the active substance and log-transformed concentration.

No	Model with Selected Variables	AIC
1	log%dermal.absorption.mean ~ mol.weight + Log.Kow + Molar.refractivity + Hbond.acceptor + Hbond.donor + TPSA + Van.der.Waals.Surface.Area + LogD + molecular.Polarizability + Rezeptor.Medium + LogConc	−766.56
2	log%dermal.absorption.mean ~ mol.weight + Log.Kow + Molar.refractivity + Hbond.acceptor + Hbond.donor + TPSA + Van.der.Waals.Surface.Area + LogD + molecular.Polarizability + LogConc	−768.55
3	log%dermal.absorption.mean ~ Log.Kow + Molar.refractivity + Hbond.acceptor + Hbond.donor + TPSA + Van.der.Waals.Surface.Area + LogD + molecular.Polarizability + LogConc	−770.49
4	log%dermal.absorption.mean ~ Log.Kow + Hbond.acceptor + Hbond.donor + TPSA + Van.der.Waals.Surface.Area + LogD + molecular.Polarizability + LogConc	−772.38
5	log%dermal.absorption.mean ~ Log.Kow + Hbond.acceptor + Hbond.donor + TPSA + Van.der.Waals.Surface.Area + molecular.Polarizability + LogConc	−773.88
6	log%dermal.absorption.mean ~ Log.Kow + Hbond.acceptor + Hbond.donor + Van.der.Waals.Surface.Area + molecular.Polarizability + LogConc	−774.86

**Table 4:** Selected regression model (no 6) with significant parameters (cut-off specified at 0.1) carried out by using the stepwise regression with R software.

Model No 6 with Selected Variables log%dermal.absorption.mean	Degrees of Freedom	Sum of Squares	Residual Sum of Squares	AIC
Log.Kow	1	1.362	411.47	−773.72
Hbond.acceptor	1	1.847	411.95	−772.61
Hbond.donor	1	12.286	422.39	−748.96
Van.der.Waals.Surface.Area	1	13.574	423.68	−746.08
molecular.Polarizability	1	14.937	425.04	−743.05
LogConc	1	186.401	596.51	−422.79

## Data Availability

The original contributions presented in the study were included in the article and Supplementary Material. Further inquiries can be directed to the corresponding authors.

## Supplementary Materials

The following supporting information can be downloaded at: https://www.mdpi.com/article/10.3390/toxics12040248/s1. Table S1: dataset of in vitro dermal absorption studies with analysed parameters. Table S2: Dependence of percentage dermal absorption on dilution (=tested concentrations).

## Abbreviations

A(O)EL: Acceptable (Operator) Exposure Level; AI: experimental solution of active substances in solvent, AIC: Akaike information criterion; Balaban index: Balaban distance connectivity of the molecule (average distance sum connectivity); BfR: German Federal Institute for Risk Assessment; BSA: bovine serum albumin; CB: bait concentrate; CS: capsule suspension; DC: dispersible concentrate; DP: dustable powder; EC: emulsifiable concentrate; ED: electrochargeable liquid; EFSA: European Food Safety Authority; EU: European Union; EW: emulsion/oil in water; FAO: Food and Agriculture Organisation of the United Nations; FL: water-based flowable concentrate; FS: flowable concentrate for seed treatment; GEL/GD: gel for direct application; GR: granule; HbA: hydrogen bond acceptor count; HbD: hydrogen bond donor count; log%DA: logarithm (base 10) of the percentage dermal absorption; logConc: logarithm (base 10) transformed concentration; LogD: logarithm (base 10) of the distribution coefficient; LogKOW: logarithm of octanol/water partition coefficient; LogP: logarithm (base 10) of the partition coefficient; ME: microemulsion; MW: molecular weight; NA: not assigned; OD: oil-based suspension concentrate; OECD: Organisation for Economic Co-operation and Development; OF: oil miscible flowable concentrate (oil miscible suspension); pKa: molar refractivity, negative base-10 logarithm of the acid dissociation constant; pKa: negative logarithm (base 10) of the acid dissociation constant; PS: seed coated with a pesticide; RAC: Risk Assessment Committee; RB: bait, ready for use; SC: suspension concentrate; SCoPAFF: Standing Committee on Plants, Animals, Food and Feed; SG: water-soluble granules; SL: soluble concentrate; SMILES: simplified molecular-input line-entry system; SP: water-soluble powder; TG: Test Guideline; TpSA: topological polar surface area; vdW SA: Van der Waals surface area; WG: water-dispersible granules; WHO: World Health Organisation; WP: wettable powder; WS: water dispersible powder for slurry seed treatment; ZC: mixture of capsule suspension and suspension concentrate.
