# Supplementary material for: In Vitro Human Dermal Absorption Studies on Pesticides in Complex Mixtures: Investigation of Guidance Criteria and Possible Impact Parameters
Source: Toxics. 2024 Mar 28;12(4):248. doi: 10.3390/toxics12040248 (PMC11054108; doi:10.3390/toxics12040248)
Supplement: Supplementary file 1 [file toxics-12-00248-s001.zip › Supplemental material_S 2 Dependence of percentage dermal absorption on dilution.pdf]

| Active substance | Tested concentrations [g/L] 1 | 2 | 3 | mean % DA 1 | 2 | 3 | area dose µg/cm² test 1 | 2 | 3 | mean % DA vs. concentration (inverse/non-inverse) |
|---|---|---|---|---|---|---|---|---|---|---|
| Glyphosate | 346.6 | 20.8 | 0.06 | 0.48 | 2.72 | 3.81 | 3470 | 210 | 100 | inverse |
| Cymoxanil | 47.8 | 0.6 | 0.06 | 0.98 | 7.52 | 27.65 | 480 | 10 | 0.6 | inverse |
| Metalaxyl-M | 514.3 | 9.8 | 1.06 | 7.61 | 66.58 | 58.5 | 40200 | 770 | 80 | non-inverse |
| Dimethenamid | 40 | 4 | 0.4 | 22.64 | 22.64 | 48.5 | 530 | 60 | 10 | inverse |
| Pyraclostrobin | 37.5 | 7.5 | 1.5 | 25.13 | 21.82 | 20.22 | 380 | 80 | 20 | non-inverse |
| Picolinafen | 377 | 0.508 | 0.13 | 0.81 | 2.72 | 10.93 | 3540 | 4.8 | 1.2 | inverse |
| Profenofos | 510.17 | 2.59 | 0.51 | 5.86 | 27.54 | 36.7 | 40080 | 210 | 40 | inverse |
| Famoxadone | 95 | 7.5 | 0.75 | 9.25 | 27.67 | 55.13 | 2500 | 190 | 20 | inverse |
| Flusilazole | 50 | 5 | 0.5 | 9.27 | 43.43 | 56.5 | 500 | 50 | 10 | inverse |
| Quinmerac | 100 | 1 | 0.38 | 1.31 | 0.99 | 0.92 | 1000 | 10 | 3.8 | non-inverse |
| Fluoxastrobin | 100 | 1.25 | 0.15 | 0.88 | 1.7 | 3.89 | 1 | 10 | 1.5 | inverse |
| Myclobutanil | 200 | 0.48 | 0.048 | 6.37 | 23.9 | 25.95 | 1880 | 4.9 | 0.5 | inverse |
| Fenhexamid | 500 | 5 | 0.375 | 0.11 | 1.07 | 6.71 | 5000 | 50 | 3.8 | inverse |
| Dimethomorph | 228.7 | 1.87 | 0.179 | 2.37 | 5.86 | 16.75 | 2020 | 20 | 1.8 | inverse |
| Boscalid | 107.5 | 1.71 | 0.23 | 0.23 | 1.05 | 3.53 | 1010 | 20 | 2.1 | inverse |
| Folpet | 500 | 7.5 | 1.25 | 0.92 | 17.47 | 14.34 | 5000 | 80 | 10 | non-inverse |
| Difenoconazole | 250 | 1.25 | 0.05 | 1.12 | 12.89 | 15.04 | 2350 | 10 | 0.5 | inverse |
| Prochloraz | 270 | 53.36 | 2.11 | 0.39 | 2.42 | 11.1 | 2700 | 530 | 20 | inverse |
| Folpet | 600 | 7.5 | 1.25 | 0.15 | 14.56 | 10.47 | 6000 | 80 | 10 | non-inverse |
| Fenpropidin | 371 | 3.66 | 0.953 | 2.47 | 47.88 | 50.93 | 3710 | 40 | 10 | inverse |
| Difenoconazole | 106 | 1.05 | 0.263 | 17.37 | 14.48 | 1.06 | 1060 | 10 | 2.6 | non-inverse |
| Mandipropamid | 250 | 0.67 | 0.14 | 0.13 | 0.68 | 1.85 | 2.540 | 10 | 1.4 | inverse |
| Dimethenamid-P | 200 | 5 | 1.25 | 4.21 | 14.9 | 16.12 | 2050 | 50 | 10 | inverse |
| Glyphosate | 480 | 28.7 | 2.4 | 0.17 | 0.27 | 0.83 | 4910 | 300 | 30 | inverse |
| Pyraclostrobin | 133.9 | 2.01 | 0.157 | 0.1 | 2.04 | 8.07 | 1310 | 20 | 1.7 | inverse |
| Tetraconazole | 42.3 | 0.42 | 0.04 | 2.67 | 36.74 | 46.02 | 380 | 4 | 0.4 | inverse |
| Isopyrazam | 82.64 | 0.76 | 0.2 | 2.99 | 59.01 | 71.38 | 800 | 10 | 2 | inverse |
| Cyproconazole | 104.83 | 0.95 | 0.25 | 0.74 | 17.66 | 39.78 | 100 | 10 | 2.5 | inverse |
| Azoxystrobin | 104.83 | 1 | 0.24 | 0.37 | 4.82 | 6.35 | 100 | 10 | 2.5 | inverse |
| Prosulfocarb | 620 | 25 | 8.4 | 8.18 | 34.54 | 46.2 | 6200 | 250 | 80 | inverse |
| Fluxapyroxad | 62.5 | 0.833 | 0.278 | 0.96 | 2.87 | 4.04 | 630 | 10 | 2.8 | inverse |
| Metconazole | 45 | 0.6 | 0.2 | 0.57 | 13.41 | 21.61 | 420 | 10 | 1.9 | inverse |
| Fluxapyroxad | 287 | 19 | 1.84 | 0.74 | 0.4 | 1.04 | 2690 | 180 | 20 | non-inverse |
| Fenamidone | 75 | 1 | 0.375 | 0.4 | 2.13 | 1.18 | 750 | 10 | 3.8 | non-inverse |
| Propamocarb HCl | 375 | 5 | 0.75 | 0.37 | 5.93 | 6.99 | 3750 | 50 | 10 | inverse |
| Cyproconazole | 125.73 | 1.31 | 0.34 | 0.09 | 5.4 | 5.56 | 1230 | 10 | 3.3 | inverse |
| Isopyrazam | 83.48 | 0.62 | 0.18 | 0.27 | 12.7 | 23.32 | 840 | 10 | 2.1 | inverse |
| Imidacloprid | 700 | 2 | 0.07 | 0.1 | 0.49 | 6.94 | 3600 | 150 | 20 | inverse |
| Fluxapyroxad | 75 | 1.12 | 0.2 | 0.26 | 8.22 | 9.5 | 760 | 10 | 1.9 | inverse |
| Pyraclostrobin | 150 | 2.24 | 0.375 | 0.31 | 8.9 | 9.83 | 1410 | 20 | 4.1 | inverse |
| Isopyrazam | 125 | 0.83 | 0.125 | 0.11 | 17.52 | 21.22 | 1320 | 10 | 1.2 | inverse |
| Azoxystrobin | 200 | 1.3 | 0.2 | 0.1 | 5.38 | 16.02 | 2050 | 10 | 2.1 | inverse |
| Cymoxanil | 225 | 0.375 | 0.094 | 0.21 | 10.34 | 20.95 | 2250 | 3.8 | 0.9 | inverse |
| Difenoconazole | 114 | 0.61 | 0.08 | 0.43 | 24.18 | 29.25 | 1140 | 10 | 0.8 | inverse |
| Isopyrazam | 122 | 1.35 | 0.1 | 1.55 | 3.86 | 9.1 | 1220 | 10 | 1 | inverse |

| | | | | | | | | | | |
|---|---|---|---|---|---|---|---|---|---|---|
| Azoxystrobin | 200 | 2 | 0.5 | 0.58 | 3.67 | 4.59 | 1910 | 20 | 10 | inverse |
| Isopyrazam | 125 | 1.25 | 0.3 | 0.77 | 5.24 | 3.99 | 1150 | 10 | 3.2 | non-inverse |
| Zoxamide | 121 | 1.1 | 0.1 | 0.63 | 6.08 | 5.76 | 1210 | 10 | 1 | non-inverse |
| Isopyrazam | 62.5 | 1.25 | 0.3 | 0.42 | 5.4 | 13.73 | 630 | 10 | 3.2 | inverse |
| Propiconazole | 62.5 | 1.25 | 0.3 | 0.91 | 5.18 | 16.93 | 630 | 10 | 3.1 | inverse |
| Thiophanate-methyl | 350 | 0.225 | 0.15 | 0.81 | 16.58 | 17.64 | 3620 | 2.4 | 1.5 | inverse |
| Tebuconazole | 103 | 0.1 | 0.068 | 1.84 | 22.72 | 23.12 | 1050 | 1 | 0.7 | inverse |
| Dimethomorph | 250 | 5 | 0.5 | 2.37 | 3.33 | 10.02 | 2960 | 50 | 10 | inverse |
| Napropamide | 450 | 22.5 | 0.9 | 0.7 | 6.51 | 13.43 | 4500 | 230 | 10 | inverse |
| Metribuzin | 175 | 52.5 | 26.25 | 0.65 | 2.28 | 1.19 | 7000 | 530 | 260 | non-inverse |
| Pymetrozine | 163 | 1.05 | 0.259 | 0.05 | 1.19 | 2.27 | 1630 | 1.1 | 0.3 | inverse |
| Epoxiconazole | 62.5 | 1.25 | 0.208 | 0.99 | 9.4 | 47.61 | 630 | 10 | 2.1 | inverse |
| Fluxapyroxad | 62.5 | 1.25 | 0.208 | 1 | 2.7 | 7.98 | 630 | 10 | 2.1 | inverse |
| Fluopyram | 125 | 1.5 | 0.25 | 0.17 | 8.53 | 9.73 | 1160 | 890 | 10 | inverse |
| Mandipropamid | 250 | 1 | 0.125 | 0.09 | 2.6 | 6.05 | 2190 | 10 | 1.2 | inverse |
| Propiconazole | 231 | 0.56 | 0.06 | 34.57 | 23.57 | 33.22 | 2170 | 10 | 0.6 | inverse |
| Metiram | 143 | 20 | 2.6 | 4.15 | 13.55 | 33.11 | 1430 | 200 | 30 | inverse |
| Metiram | 143 | 20 | 2.6 | 4.18 | 13.55 | 33.11 | 1430 | 200 | 30 | inverse |
| Oxydemeton-methyl | 255.5 | 4.26 | 0.44 | 38.42 | 31.43 | 59.09 | 3990 | 70 | 10 | inverse |
| Metribuzin | 186.78 | 41.88 | 3.95 | 7.83 | 21.47 | 84.84 | 1870 | 420 | 40 | inverse |
| Oxydemeton-methyl | 192.5 | 3.7 | 0.38 | 6.22 | 25.4 | 27.07 | 3010 | 60 | 10 | inverse |
| Methamidophos | 600 | 60 | 2 | 3.63 | 12.33 | 37.3 | 9370 | 940 | 30 | inverse |
| Tolylfluanid | 63.8 | 7.1 | 0.68 | 82.58 | 52.38 | 56.9 | 640 | 70 | 10 | non-inverse |
| Quizalofop-P-tefuryl | 40 | 0.5 | 0.25 | 2.29 | 16.42 | 18.97 | 430 | 10 | 2.7 | inverse |
| Imidacloprid | 200 | 0.7 | 0.07 | 0.3 | 8.04 | 6.01 | 2000 | 10 | 0.7 | non-inverse |
| Aviglycine hydrochloride | 25 | 2.5 | 0.125 | 1.54 | 0.81 | 1.72 | 250 | 30 | 1.3 | non-inverse |
| Boscalid | 100 | 10 | 1 | 2.09 | 21.3 | 66.61 | 810 | 90 | 10 | inverse |
| Cyproconazole | 60 | 1 | 0.12 | 23.87 | 53.11 | 71.82 | 640 | 10 | 1.2 | inverse |
| Cloquintocet-methyl | 12.5 | 1.25 | 0.125 | 0.63 | 12.96 | 43.34 | 1110 | 100 | 10 | inverse |
| Flusilazole | 400 | 50 | 5 | 18.19 | 36.31 | 67.33 | 4000 | 500 | 50 | inverse |
| Imidacloprid | 350 | 70 | 0.5 | 0.04 | 0.11 | 5.01 | 3500 | 700 | 10 | inverse |
| Trifloxystrobin | 131.39 | 18.92 | 3.02 | 27.3 | 3.66 | 5.02 | 10270 | 1480 | 240 | inverse |
| Dimoxystrobin | 30 | 6 | 1.2 | 3.16 | 12.06 | 4.43 | 300 | 60 | 10 | non-inverse |
| Chlorothalonil | 745 | 7.44 | 0.92 | 0.1 | 0.61 | 2.25 | 3700 | 40 | 4.6 | inverse |
| Terbuthylazine | 166.65 | 16.67 | 0.51 | 0.32 | 0.34 | 1.12 | 3500 | 500 | 1.2 | inverse |
| Azoxystrobin | 93.5 | 1.039 | 0.134 | 6.1 | 3.1 | 0.17 | 940 | 10 | 1.3 | non-inverse |
| Folpet | 500 | 5.5 | 0.7 | 0.43 | 2.34 | 8.8 | 5000 | 60 | 10 | inverse |
| Cyfluthrin, beta- | 80 | 40 | 11.4 | 0.04 | 0.14 | 0.33 | 700 | 390 | 110 | inverse |
| Epoxiconazole | 125 | 1.25 | 0.313 | 1.9 | 11.17 | 11.24 | 1260 | 10 | 3.6 | inverse |
| Terbuthylazine | 326 | 3.75 | 1.63 | 0.94 | 5.78 | 12.52 | 2930 | 40 | 20 | inverse |
| Metolachlor, S- | 326.5 | 8.92 | 2.26 | 35.39 | 27.72 | 1.99 | 3270 | 90 | 20 | non-inverse |
| Bromoxynil octanoate | 327.6 | 4.913 | 1.23 | 0.9 | 7.86 | 7.18 | 3280 | 50 | 10 | inverse |
| Diflufenican | 171 | 1.5 | 0.3 | 0.09 | 5.02 | 9.83 | 1700 | 20 | 3 | inverse |
| Metribuzin | 64 | 0.56 | 0.11 | 0.05 | 12.19 | 19.6 | 630 | 10 | 1.2 | inverse |
| Clothianidin | 100 | 50 | 11.1 | 0.04 | 0.09 | 1.25 | 830 | 450 | 90 | inverse |

| | | | | | | | | | | |
|---|---|---|---|---|---|---|---|---|---|---|
| Clothianidin | 600 | 102 | 22.6 | 0.03 | 0.2 | 0.26 | 4990 | 450 | 90 | inverse |
| Flufenacet | 171 | 1.5 | 0.3 | 0.16 | 12.85 | 16.12 | 1640 | 10 | 3 | inverse |
| Triasulfuron | 66.7 | 0.15 | 0.075 | 0.03 | 1.12 | 2.97 | 670 | 1.7 | 0.9 | inverse |
| Fenoxycarb | 250 | 0.75 | 0.05 | 0.53 | 10.53 | 46.35 | 2500 | 10 | 0.5 | inverse |
| Spirotetramat | 150 | 1.5 | 0.05 | 0.24 | 2.68 | 3.8 | 1500 | 20 | 0.5 | inverse |
| Folpet | 500 | 7.5 | 1.2 | 0.92 | 17.47 | 14.34 | 5000 | 75 | 12.5 | non-inverse |
| Flupyradifurone | 200 | 0.625 | 0.1 | 0.2 | 1.99 | 4.73 | 2000 | 6.3 | 1 | inverse |
| Sedaxane | 500 | 25 | 2.5 | 0.07 | 0.55 | 0.57 | 5000 | 250 | 25 | inverse |
| Dimethazone (Command, Clomazone) | 360 | 2.44 | 0.09 | 0.6 | 11.35 | 18.39 | 3600 | 24.4 | 0.9 | inverse |
| Isopyrazam | 100 | 1 | 0.25 | 0.74 | 15.93 | 39.72 | 1000 | 10 | 2.5 | inverse |
| Spirotetramat | 100 | 0.75 | 0.075 | 0.05 | 1.98 | 5.23 | 1000 | 7.5 | 0.8 | inverse |
| Difenoconazole | 125 | 0.625 | 0.083 | 0.45 | 24.25 | 29.4 | 1250 | 6.3 | 0.8 | inverse |
| Prothioconazole | 100 | 1.25 | 0.25 | 4.68 | 16.74 | 27.26 | 1000 | 12.5 | 2.5 | inverse |
| Glyphosate | 360 | 36 | 2.2 | 0.06 | 0.18 | 0.05 | 3600 | 360 | 22 | non-inverse |
| Imazalil | 25 | 16.7 | 12.5 | 2.7 | 3.4 | 4.37 | 250 | 167 | 125 | inverse |
| Fluxapyroxad | 75 | 0.5 | 0.25 | 0.61 | 0.78 | 1.29 | 750 | 5 | 2.5 | inverse |
| Difenoconazole | 50 | 0.33 | 0.167 | 1.11 | 6.15 | 8.88 | 500 | 3.3 | 1.7 | inverse |
| Iprovalicarb | 90 | 1.2 | 0.07 | 0.21 | 2.6 | 10.84 | 450 | 12 | 0.7 | inverse |
| Pyrimethanil | 336 | 2.688 | 0.538 | 0.12 | 11.92 | 38.17 | 94915 | 759.3 | 151.9 | inverse |
| Napropamide | 450 | 22.5 | 0.9 | 0.7 | 6.51 | 13.43 | 4500 | 225 | 9 | inverse |
| Cyantraniliprole | 200 | 0.749 | 0.25 | 0.01 | 0.2 | 0.27 | 2000 | 7.5 | 2.5 | inverse |
| Mandipropamid | 250 | 1 | 0.125 | 0.09 | 2.6 | 5.81 | 2500 | 10 | 1.3 | inverse |
| Difenoconazole | 125 | 0.625 | 0.083 | 0.45 | 24.25 | 29.4 | 1250 | 6.3 | 0.8 | inverse |
| Isopyrazam | 125 | 1.25 | 0.313 | 0.47 | 11.5 | 17.29 | 1250 | 12.5 | 3.1 | inverse |
| Spinetoram | 25 | 0.25 | 0.025 | 1.08 | 3.59 | 6.01 | 250 | 2.5 | 0.3 | inverse |
| Trifloxystrobin | 250 | 0.2 | 0.033 | 0.03 | 0.58 | 9.98 | 2500 | 2 | 0.3 | inverse |
| Flufenacet | 500 | 1.2 | 0.25 | 0.18 | 4.19 | 21.71 | 5000 | 12 | 2.5 | inverse |
| Fludioxonil | 250 | 0.83 | 0.125 | 0.03 | 3 | 5.95 | 2500 | 8.3 | 1.3 | inverse |
